# Evidence of Pathogen-Induced Immunogenetic Selection across the Large Geographic Range of a Wild Seabird

**DOI:** 10.1093/molbev/msaa040

**Published:** 2020-02-25

**Authors:** Hila Levy, Steven R Fiddaman, Juliana A Vianna, Daly Noll, Gemma V Clucas, Jasmine K H Sidhu, Michael J Polito, Charles A Bost, Richard A Phillips, Sarah Crofts, Gary D Miller, Pierre Pistorius, Francesco Bonnadonna, Céline Le Bohec, Andrés Barbosa, Phil Trathan, Andrea Raya Rey, Laurent A F Frantz, Tom Hart, Adrian L Smith

**Affiliations:** m1 Department of Zoology, University of Oxford, Oxford, United Kingdom; m2 Departamento de Ecosistemas y Medio Ambiente, Pontificia Universidad Católica de Chile, Macul, Santiago, Chile; m3 Departamento de Ciencias Ecológicas, Instituto de Ecología y Biodiversidad, Universidad de Chile, Santiago, Chile; m4 Cornell Atkinson Center for a Sustainable Future, Cornell University, Ithaca, NY; m5 Cornell Lab of Ornithology, Cornell University, Ithaca, NY; m6 Department of Oceanography and Coastal Sciences, Louisiana State University, Baton Rouge, LA; m7 Centre d’Etudes Biologiques de Chizé (CEBC), UMR 7372 du CNRS‐Université de La Rochelle, Villiers‐en‐Bois, France; m8 British Antarctic Survey, Cambridge, United Kingdom; m9 Falklands Conservation, Stanley, Falkland Islands, United Kingdom; m10 Microbiology and Immunology, PALM, University of Western Australia, Crawley, Western Australia, Australia; m11 DST/NRF Centre of Excellence at the Percy FitzPatrick Institute for African Ornithology, Department of Zoology, Nelson Mandela University, Port Elizabeth, South Africa; m12 CEFE UMR 5175, CNRS, Université de Montpellier, Université Paul-Valéry Montpellier, EPHE, Montpellier, France; m13 Université de Strasbourg, CNRS, IPHC UMR 7178, Strasbourg, France; m14 Département de Biologie Polaire, Centre Scientifique de Monaco, Monaco, Principality of Monaco; m15 Museo Nacional de Ciencias Naturales, Departamento de Ecología Evolutiva, CSIC, Madrid, Spain; m16 Centro Austral de Investigaciones Científicas – Consejo Nacional de Investigaciones Científicas y Técnicas (CADIC-CONICET), Ushuaia, Tierra del Fuego, Argentina; m17 Instituto de Ciencias Polares, Ambiente y Recursos Naturales, Universidad Nacional de Tierra del Fuego, Ushuaia, Tierra del Fuego, Argentina; m18 Wildlife Conservation Society, Buenos Aires, Argentina; m19 School of Biological and Chemical Sciences, Queen Mary University of London, London, United Kingdom

**Keywords:** immunogenetics, positive selection, Toll-like receptors, pathogen-mediated selection, Antarctica and Southern Ocean, Gentoo penguin

## Abstract

Over evolutionary time, pathogen challenge shapes the immune phenotype of the host to better respond to an incipient threat. The extent and direction of this selection pressure depend on the local pathogen composition, which is in turn determined by biotic and abiotic features of the environment. However, little is known about adaptation to local pathogen threats in wild animals. The Gentoo penguin (*Pygoscelis papua*) is a species complex that lends itself to the study of immune adaptation because of its circumpolar distribution over a large latitudinal range, with little or no admixture between different clades. In this study, we examine the diversity in a key family of innate immune genes—the Toll-like receptors (TLRs)—across the range of the Gentoo penguin. The three TLRs that we investigated present varying levels of diversity, with *TLR4* and *TLR5* greatly exceeding the diversity of *TLR7*. We present evidence of positive selection in *TLR4* and *TLR5*, which points to pathogen-driven adaptation to the local pathogen milieu. Finally, we demonstrate that two positively selected cosegregating sites in *TLR5* are sufficient to alter the responsiveness of the receptor to its bacterial ligand, flagellin. Taken together, these results suggest that Gentoo penguins have experienced distinct pathogen-driven selection pressures in different environments, which may be important given the role of the Gentoo penguin as a sentinel species in some of the world’s most rapidly changing environments.

## Introduction

All organisms are challenged by pathogens in their surrounding environments, but it is clear that the pathogen pressure can vary by location. Similarly, to free-living metazoans, a latitudinal species-richness gradient has been identified in several parasitic and pathogenic taxa, which may be driven by temperature and other abiotic and biotic factors (Rohde and Heap 1998; Guernier et al. 2004; Dionne et al. 2007). Given this gradient in pathogen pressure, it follows that natural selection on the host will favor distinct immune phenotypes in different environments, as suggested by major histocompatibility complex II genetic diversity patterns in Humboldt penguins associated with higher pathogen diversity in lower latitudes (Sallaberry-Pincheira et al. 2015, 2016). In our study, we sought to test the hypothesis that pathogen-driven selection can drive distinct patterns of host immune system genotype and phenotype, using the Gentoo penguin (*Pygoscelis papua* ssp.) as a model species complex.

The Gentoo penguin complex (Vianna et al. 2017; Clucas et al. 2018) is ideally suited for investigating pathogen-driven selection on the immune system. Firstly, it has a circumpolar range, spanning the largest latitudinal range of any penguin species, between 46° S and 66° S, with breeding colonies in most of the Southern Ocean’s sub-Antarctic islands, as well as the islands off Tierra del Fuego in South America, South Georgia, the Scotia Arc, and the Western Antarctic Peninsula (Stonehouse 2008). Secondly, population monitoring of the species has shown it to be growing at the southern end of its range (Lynch et al. 2012), with highly fluctuating changes over time in colonies in the South Atlantic and Indian Oceans (Lescroel and Bost 2006; Trathan et al. 2007). Thirdly, the Gentoo penguin is a highly philopatric seabird known to remain close to its breeding colonies year round (Trivelpiece et al. 1987; Wilson et al. 1998; Clausen and Putz 2003; Hinke et al. 2017), limiting gene flow across breeding regions (Levy et al. 2016; Vianna et al. 2017; Clucas et al. 2018).

Furthermore, across its range, Gentoo penguins overlap with (and occasionally co-occur in mixed colonies with) King (*Aptenodytes patagonicus*), Magellanic (*Spheniscus magellanicus*), Macaroni (*Eudyptes chrysolophus*), and Southern Rockhopper penguins (*Eudyptes chrysocome*) in sub-Antarctic colonies, as well as congeneric Adélie (*P. adeliae*) and Chinstrap penguins (*P. antarcticus*) in its Antarctic range. Gentoo penguin colonies are also frequented by a number of flying birds with vast ranges, including albatrosses and petrels (Order: Procellariiformes), as well as predator-scavengers like skuas (genus *Stercorarius*) and sheathbills (genus *Chionis*) that could introduce and/or spread novel avian pathogens. Levels of human interaction also vary across the range, from permanent settlements with livestock present near colonies in the Falkland/Malvinas Islands, to seasonal or year-round scientific research stations in sub-Antarctic and Antarctic colonies, and an increasing presence of Antarctic tourism. Differences in sympatric interactions with other species across the range of the Gentoo penguin are likely to result in different pathogen challenges and therefore different selective pressures.

To investigate genetic diversity across the immune system, many immunogenetic studies on penguins have focused on the major histocompatibility complex (Tsuda et al. 2001; Bollmer et al. 2007; Knafler et al. 2012; Sallaberry-Pincheira et al. 2016). Increasingly, however, the Toll-like receptors (TLRs) are recognized as important monogenic determinants of disease-resistance phenotypes, and are therefore important operands for natural selection (Grueber et al. 2014). Toll-like receptors are the best-studied family of pattern-recognition receptors in the vertebrate innate immune system, representing the front line of detection of pathogen challenge (Kawai and Akira 2006). TLRs respond to highly conserved microbe-associated molecular patterns (MAMPs) that are structurally conserved in large groups of pathogens. Upon binding of a MAMP, TLRs undergo dimerization and initiate an intracellular signaling cascade that culminates in the production of antipathogen effector molecules (Akira et al. 2001; Botos et al. 2011).

Vertebrates have six major families of TLRs which are typically conserved across evolutionary time to retain specificity for a particular MAMP or family of MAMPs. In most avian species, there are ten recognized TLRs (Roach et al. 2005; Boyd et al. 2007; Brownlie and Allan 2011). Of these, TLR4 and TLR5 respond to the bacterial agonists lipopolysaccharide and flagellin, respectively, whereas TLR7 responds to single-stranded RNA of viruses in the endosomal compartment (Chow et al. 1999; Gewirtz et al. 2001; Lund et al. 2004).

**Fig. 1.**
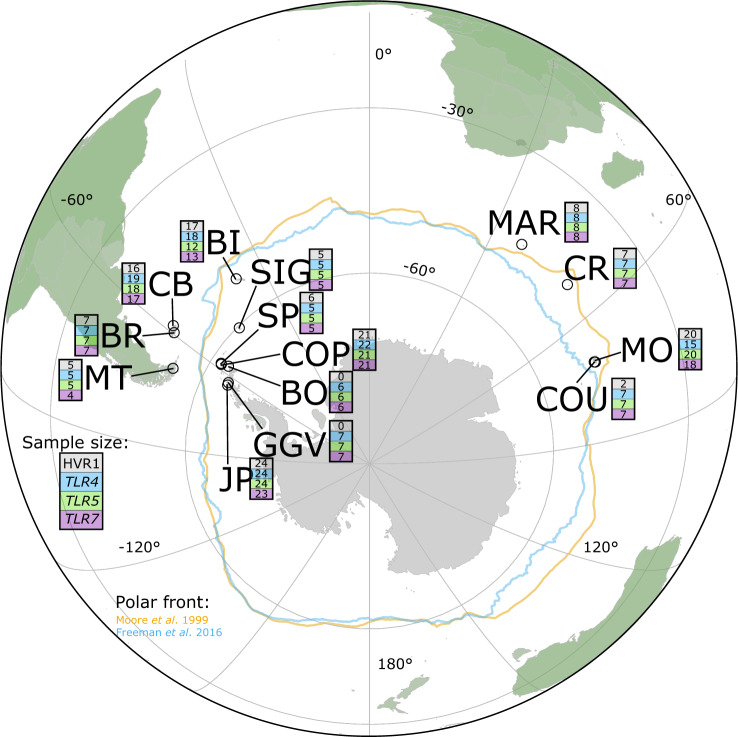
Locations and per-locus sample sizes for each sampled Gentoo penguin colony in the Southern Ocean and Antarctica. Solid-colored lines represent the reported position of the Polar Front, based on the analyses of Freeman et al. (2016) and Moore et al. (1999). Depending on the analysis, the Kerguelen and Crozet Islands can lie just north or just south of the Polar Front (Moore et al. 1999; Freeman et al. 2016). CR, Crozet Island; MAR, Marion Island; COU, Courbet Peninsula, Kerguelen; MO, Pointe du Morne, Kerguelen; CB, Cow Bay, Falkland/Malvinas Islands; BR, Bull Roads, Falkland/Malvinas Islands; BI, Bird Island, South Georgia; MT, Martillo Island, Tierra del Fuego; SIG, Signy Island, South Orkney Islands; COP, Copacabana (Admiralty Bay), King George Island, South Shetland Islands; SP, Stranger Point, King George Island, South Shetland Islands; BO, Bernardo O’Higgins Base, Western Antarctic Peninsula; GGV, Gabriel González Videla Base, Western Antarctic Peninsula; JP, Jougla Point, Western Antarctic Peninsula.

To investigate TLR diversity across the range of the Gentoo penguin complex, we sequenced the full coding sequences of *TLR4*, *TLR5*, and *TLR7*, as opposed to targeted portions of certain exons as in previous studies (Dalton, Vermaak, Roelofse, et al. 2016). These three genes represent bacterial- and viral-sensing TLRs that are present in almost all vertebrates. Samples (*n* = 155) were obtained from a broad geographic range across the range of the species (fig. 1), representing the largest geospatial scale of any immunogenetics study outside of humans. We describe patterns of diversity in TLRs that have a clear spatial component, and provide evidence that some of the diversity in TLR4 and TLR5 is driven by positive selection between different locations. We also demonstrate that two of the positively selected residues in TLR5 yield a phenotypic difference in the response of the receptor to flagellin, providing further evidence that Gentoo penguins have experienced differential pathogen-driven selection pressures in different environments.

## Results

### Amplification of TLR Genes in the Gentoo Penguin

Through successful amplification by PCR or whole-genome sequencing, we were able to confirm that Gentoo penguins have clear homologs of *TLR4*, *TLR5*, and *TLR7*, finding no evidence of gene loss or pseudogenization, as has been reported in other avian lineages for *TLR5* (Velová et al. 2018) or in African penguins for *TLR7* (Dalton, Vermaak, Roelofse, et al. 2016).

The length of the *P. papua TLR4* coding sequence matches the longest reported length (2,550 bp/849 aa) in other bird species. For *TLR5*, there is a start codon that yields an open reading frame (ORF) in line with the length of previously published *TLR5* sequences (2,589 bp/862 aa; Velová et al. 2018), but the ORF continues upstream of the putative start codon, yielding a complete ORF that is 2,643 bp/880 aa, which is 54 bp longer than other reported avian *TLR5* sequences. Both the longer and shorter ORFs respond to flagellin in our in vitro system (data not shown), suggesting both could be functional in vivo. The length of the *TLR7* coding exon, at 3,126 bp/1,042 aa, falls within the reported range of avian coding sequence lengths.

### Diversity Indices and Population Differentiation

#### Mitochondrial Hypervariable Region

**Fig. 2.**
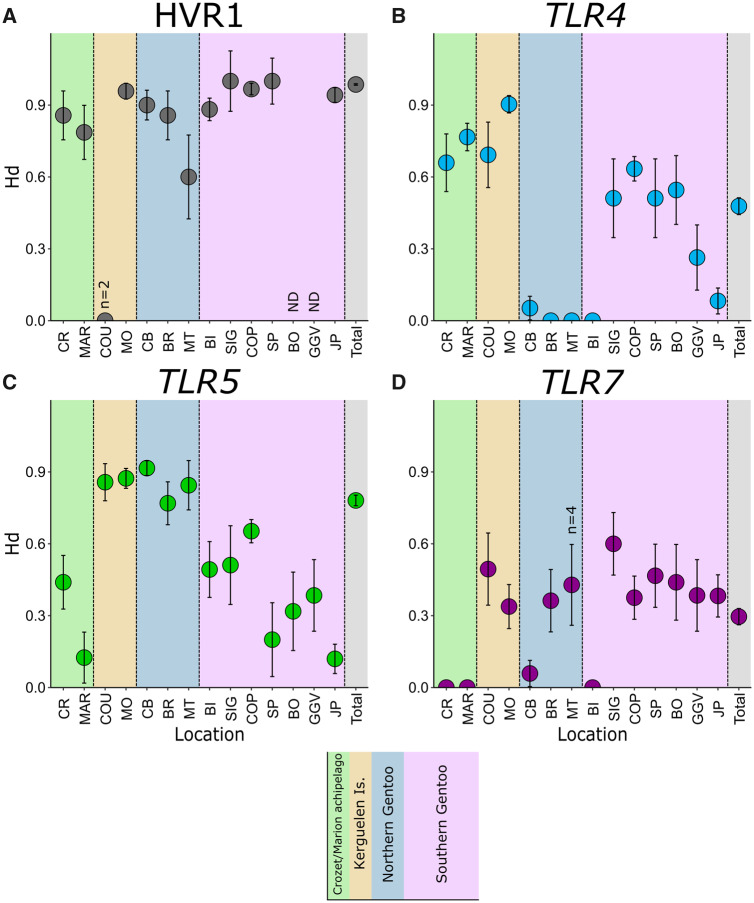
Haplotype diversity (Hd) for the hypervariable mitochondrial control region (HVR1; *A*) and three TLR loci (*B*–*D*), for all sampling sites for which data were available. All sites had *n* ≥ 5 unless otherwise indicated (ND, no data). Location abbreviations are the same as in previous figure.

All colonies with more than two sampled individuals presented high levels of mitochondrial hypervariable region 1 (HVR1) haplotype diversity (*H*_d_ = 0.60–1.00; fig. 2 and supplementary table S1, Supplementary Material online) as obtained in DnaSP 6.12.10 (Rozas et al. 2017). Differentiation between colonies at this locus was significant and in line with previous data for this species (see supplementary table S2*D*, Supplementary Material online), with four clearly differentiated clades obtained through population-level analyses in Arlequin v3.5.1.3 (Excoffier and Lischer 2010): 1) a southern clade consisting of colonies South of the Polar Front in South Georgia, the South Orkneys, the South Shetlands, and Western Antarctic Peninsula; 2) a South American/Falklands/Malvinas clade; 3) a Kerguelen clade; and 4) a North Indian Ocean clade (Marion and Crozet Islands).

**Fig. 3.**
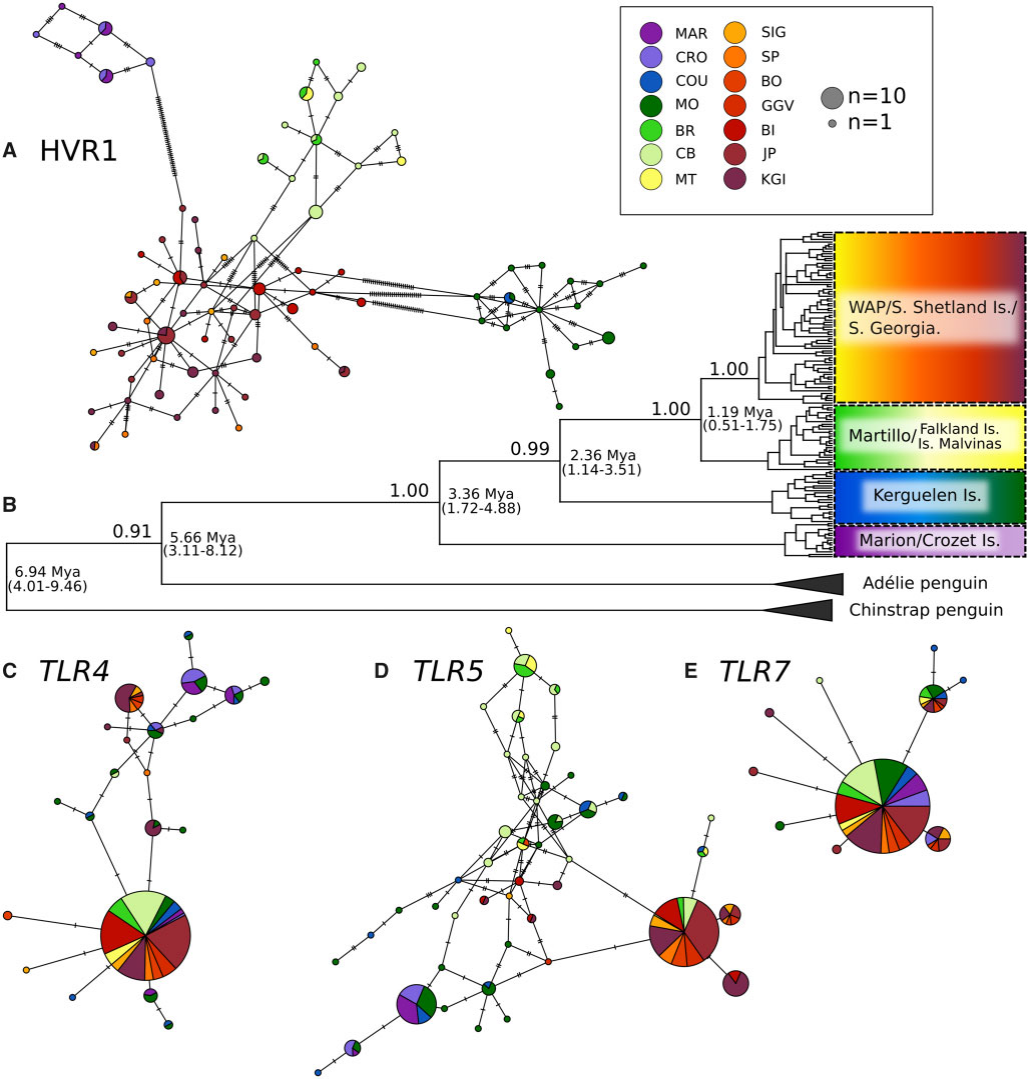
Minimum spanning haplotype networks for mtDNA HVR1 (*A*), *TLR4* (*C*), *TLR5* (*D*), and *TLR7* (*E*) for Gentoo penguin colonies, along with mtDNA HVR1 maximum clade credibility tree (*B*), using congeneric penguin species as outgroups. Location abbreviations are the same as in previous figures. For minimum spanning haplotype networks, pie charts represent single haplotypes, whereas segment size refers to the contribution of individual sampled sites to the proportion of overall haplotype frequency. Size of pie charts reflects the number of individual birds with the observed haplotype. Dashes on connecting lines each denote one nucleotide change.

Our BEAST 2 phylogenetic analysis of HVR1 showed support for a division within the Gentoo penguin complex occurring ∼3.36 Ma (1.72–4.88 Ma), when the North Indian Ocean clade diverges from all others. The Kerguelen lineage diverges from the Atlantic lineages 2.36 Ma (1.14–3.51 Ma), and the populations North and South of the Polar Front within the Atlantic Ocean appear to diverge 1.19 Ma (0.51–1.75 Ma; fig. 3). Within each clade, no clear site-specific mtDNA structure was noted.

#### TLR4

For *TLR4*, 13 polymorphic sites were identified, with a total of 21 distinct phased haplotypes coding for 9 unique protein variants (supplementary table S1, Supplementary Material online). Indian Ocean colonies (CR, MAR, COU, and MO) presented the highest levels of haplotype diversity (*H*_d_ = 0.66–0.90), whereas the South American colony in Tierra del Fuego (MT) and the Falkland/Malvinas Islands (CB and BR) had very low diversity (*H*_d_ = 0.00–0.05; fig. 2). Among Gentoo penguin colonies south of the Polar Front, diversity was highest in the central colonies of SIG, COP, SP, and BO (*H*_d_ = 0.51–0.63), decreasing sharply in BI in South Georgia (*H*_d_ = 0.00), as well as southward down the Western Antarctic Peninsula (GGV and JP; *H*_d_ = 0.08–0.26).

**Fig. 4.**
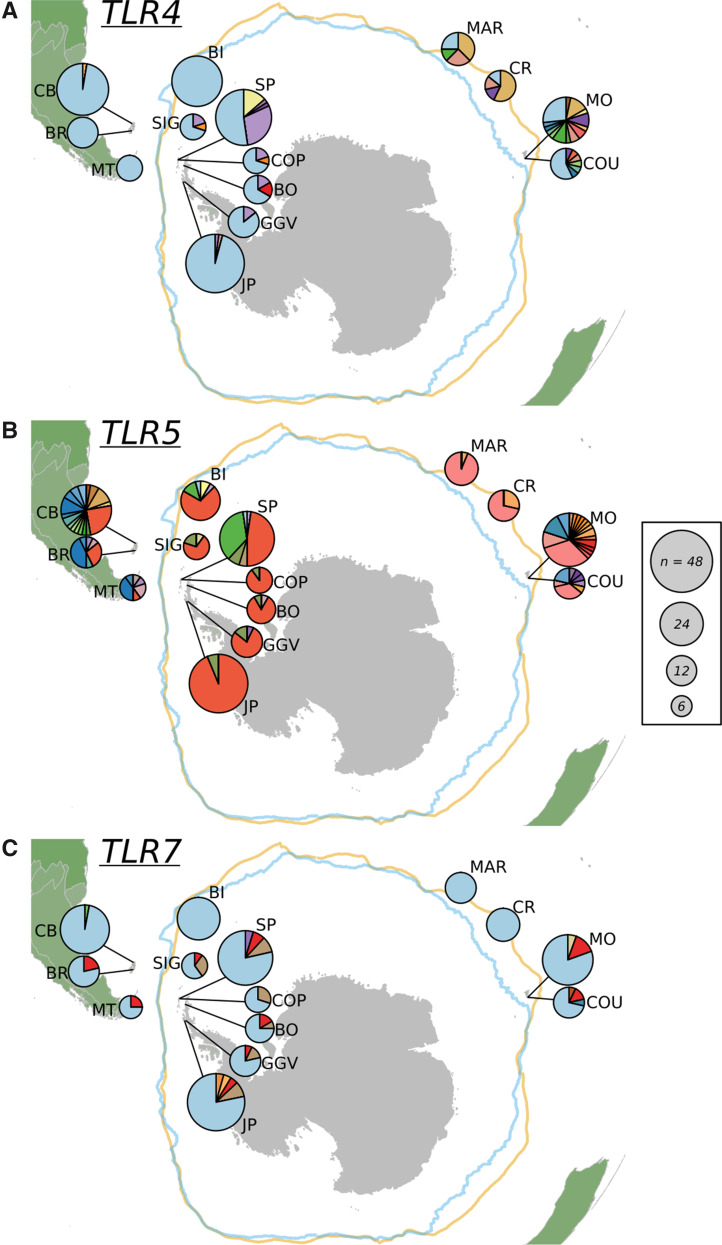
Haplotype diversity across Gentoo penguin sample populations for (*A*) *TLR4*, (*B*) *TLR5*, and (*C*) *TLR7*. For each locus, different colors represent unique haplotypes and each segment size reflects the proportion of birds in each location with that haplotype. Overall size of the pie chart reflects the number of birds sampled in each location. Location abbreviations are the same as in previous figures.

**Fig. 5.**
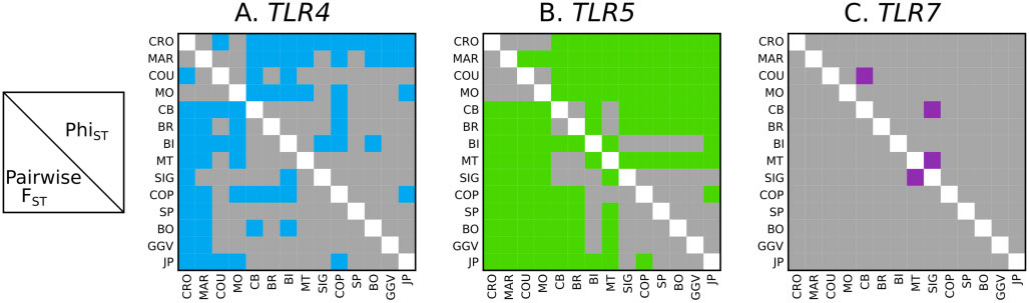
Visualization of pairings of Gentoo penguin breeding colonies, where pairwise *F*_ST_ values (below diagonal) or *Φ*_ST_ values (above diagonal) with significance *P *<* *0.01 after correction for multiple tests using SGOF+ (Carvajal-Rodríguez and de Uña-Alvarez 2011) are shown in color for (*A*) *TLR4*, (*B*) *TLR5*, and (*C*) *TLR7*. Location abbreviations are the same as in previous figures.

Comparing pairwise genetic distances (*F*_ST_ and *Φ*_ST_) for *TLR4* in Arlequin(fig. 5 and supplementary table S2*A*, Supplementary Material online), the Indian Ocean populations of Crozet and Marion differed significantly (*F*_ST_ > 0.3, *P *<* *0.01) or near-significantly (corrected *P* ∼ 0.01) from all other colonies in the Atlantic. Crozet and Marion did not differ significantly from each other (*F*_ST_ = 0.001, *P* = 0.40), whereas some haplotypes were shared between CR/MAR and the Kerguelen Island colonies of COU and MO, yielding some nonsignificant pairings among the four Indian Ocean colonies. In the Atlantic, one haplotype, also present in the Indian Ocean in lower frequencies, dominated across all the colonies (fig. 4). The central sites in the Scotia Arc (COP/SP on King George Island, Signy Island, and BO on the northern end of the Western Antarctic Peninsula) exhibited greater diversity than other Atlantic sites and contained private alleles. The overall pattern of significance for *F*_ST_ and *Φ*_ST_ comparisons can be seen in figure 5. Not surprisingly, colonies within the same island group that are in close proximity to each other (COU/MO, CB/BR, COP/SP) did not differ significantly, despite variations in sample sizes.

Hierarchical population structure was detected for *TLR4* across Gentoo penguin colonies using Arlequin (analysis of molecular variance, AMOVA; global *F*_ST_ = 0.32, *P *<* *0.0001). The proportion of variation resulting from differences among groups was 24.01% (*F*_CT_ = 0.24, *P *=* *0.003) when colonies were placed into four groups, coinciding with the four mtDNA clades: 1) Marion/Crozet archipelagos, 2) Kerguelen Is., 3) Falkland/Malvinas and Tierra del Fuego, and 4) south of the Polar Front in the Scotia Arc and Antarctic Peninsula. However, *F*_CT_ increased to 28.19% (*P *=* *0.007) when Bird Island was grouped separately from other Southern Gentoo penguin colonies, a pattern also suggested in genomic-level analyses (Clucas et al. 2018).

#### TLR5

*TLR5* was the most diverse TLR locus analyzed. Twenty polymorphic sites were identified, with a total of 46 distinct phased haplotypes coding for 32 unique protein variants (supplementary table S1, Supplementary Material online). Five Gentoo penguin colonies north of the Polar Front (COU/MO in Kerguelen, CB/BR in Falklands/Malvinas, and MT in Tierra del Fuego) exhibited the highest diversity measures (6–17 haplotypes, *H*_d_ = 0.77–0.92, fig. 2), despite differences in sample size. This is unexpected given that Martillo Island is the smallest known population of this species (*N*_c_ = 12 breeding pairs, Ghys et al. 2008), yet still maintained high diversity (five unique protein variants in a sample of *n* = 5) at this locus. Interestingly, Crozet and Marion Island colonies exhibited substantially lower genetic variation at this locus with only two haplotypes (*H*_d_ = 0.13–0.44), though these were shared with the Kerguelen colonies (*H*_d_ = 0.86–0.87; fig. 4). Southern colonies exhibited moderate diversity (*H*_d_ = 0.51–0.65), with the exception of SP in the South Shetland Islands and the Western Antarctic Peninsula at the edge of the range, with only two or three haplotypes (*H*_d_ = 0.12–0.38). Strikingly, Atlantic colonies south of the Polar Front were dominated by one haplotype found less frequently (10–28%) in the northern Atlantic colonies and completely absent from all Indian Ocean colonies. The second-most prevalent haplotype in southern colonies was completely absent from all northern colonies.

Pairwise *F*_ST_ and *Φ*_ST_ values obtained in Arlequin revealed significant clustering by clade in terms of genetic distance at the *TLR5* locus (fig. 5 and supplementary table S2*B*, Supplementary Material online). Within three of the four clades mentioned earlier, there were no significant differences (CR/MAR, COU/MO, and CB/BR/MT). The only significantly differentiated within-clade pairs lay in Gentoo penguin colonies south of the Polar Front, where Jougla Point differed significantly from COP (highest diversity among Southern Gentoo penguins) and BI (at the opposite geographic edge of this clade’s range). All other colonies differed significantly from all colonies outside their clade (*F*_ST_ range 0.1–0.88, *P *<* *0.01), with CR/COU being near-significant (*F*_ST_ = 0.13, *P *=* *0.02). This was reflected in AMOVA results, where a four-clade grouping presented a proportion of variation resulting from differences among groups of 32.70% (*F*_CT_ = 0.327, *P *<* *0.0001), and isolating Jougla Point increased the *F*_CT_ to 56.56% (*P *<* *0.0001).

#### TLR7

 

#### 

*TLR7* was the least diverse TLR locus, with nine polymorphic sites, ten phased haplotypes, and eight unique protein variants present across the study colonies (fig. 2 and supplementary table S1, Supplementary Material online). One haplotype was predominant in all colonies (frequency of 70–100%) and seven of the ten haplotypes were private alleles, only found in single colonies (fig. 4). In the Indian Ocean, Kerguelen colonies (COU/MO) had relatively more diversity (*H*_d_ = 0.34–0.50) than the Crozet and Marion Island colonies (*H*_d_ = 0.00), which had only one haplotype. In the Atlantic, South Georgia (BI) had only one haplotype (*H*_d_ = 0.00), whereas all northern Atlantic colonies (CB/BR/MT) and SP in the South Shetland Islands presented two haplotypes (*H*_d_ = 0.06–0.47). Other southern colonies contained three to four haplotypes (*H*_d_ = 0.38–0.60), whereas the southernmost colony at Jougla Point (JP) on the Western Antarctic Peninsula exhibited the most unique haplotypes (*H *=* *5; *H*_d_ = 0.38) and contained two private alleles. Unsurprisingly, only 1 of 91 pairwise comparisons between colonies were significant in terms of *F*_ST_ and 3/91 for *Φ*_ST_ (*P *<* *0.01), with no pattern to this differentiation (fig. 5 and supplementary table S2*C*, Supplementary Material online). Different AMOVA groupings yielded an among-group variation (*F*_CT_) no higher than 2.85% (four-clade grouping), further highlighting the lack of structure in this locus.

### Effect of Population Size on Diversity

Census population size was not significantly correlated to TLR haplotype diversity (see supplementary fig. S1 and tables S3 and S4, Supplementary Material online: *TLR4*, *P *=* *0.067; *TLR5*, *P *=* *0.75; *TLR7*, *P *=* *0.64).

### Isolation by Distance

Significant isolation by distance, using shortest distances by sea between colonies in a Mantel’s test, was detected in both *TLR4* (*r* = 0.515, *P *=* *0.001) and *TLR5* (*r* = 0.593, *P *=* *0.001; supplementary table S5*A*, Supplementary Material online). mtDNA HVR1 was less strongly correlated to isolation by distance (*r* = 0.312), though marginally significant (*P *=* *0.015). On the other hand, the lack of diversity and structure in the *TLR7* locus yielded no significant correlation across the range of Gentoo penguin colonies sampled.

### Analysis of Positive Selection

**Fig. 6.**
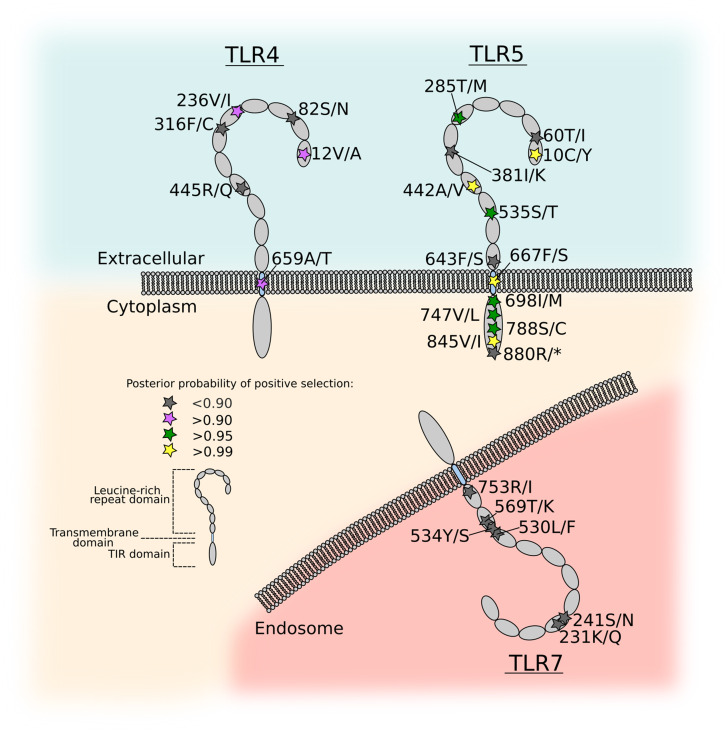
Positions of polymorphic and positively selected sites in *Pygoscelis papua* TLR4, 5, and 7. Schematic diagrams of TLRs in the extracellular (TLR4 and 5) and endosomal (TLR7) compartments show positions of amino acid variants resulting from nonsynonymous nucleotide substitutions. Variant positions are marked with stars and are colored according to the likelihood of positive selection as determined by the M2a model in the *codeml* program in PAML.

We investigated the *P. papua TLR4*, *TLR5*, and *TLR7* genes for evidence of positive selection, which could be an indicator of adaptation to local pathogen environments across the natural range of the species. Neutrality tests (Tajima’s *D* and Fu’s *F*s) did not yield observable patterns of significant deviation from neutrality across the full length of these genes (supplementary table S1, Supplementary Material online). Using a codon-specific approach, the site models in the *codeml* package of programs in PAML v4.9 (Yang 1997, 2007) were employed to test for signatures of positive selection in *P. papua* TLR loci. Nonsynonymous sites observed and analyzed are graphically depicted in their relative positions on the proteins in figure 6.

As expected, the majority of codons (*TLR4*, 98.8%; *TLR5*, 98.4%) were predicted to be under purifying selection with the ratio of nonsynonymous to synonymous substitutions (d*N*/d*S*) being <1. Interestingly, 1.2% (*TLR4*) or 1.6% (*TLR5*) of codons in the alignment were found to be positively selected using M2a, and similar frequencies, 1.2% (*TLR4*) or 1.7% (*TLR5*), were found using M8. For *TLR7*, 99.9% of sites were found to be under purifying selection, whereas the remaining 0.1% was predicted to be under neutral selection. We investigated whether models that permit positive selection were a significantly better fit to the multiple alignments than models where d*N*/d*S* ≯1 by performing likelihood ratio tests between pairs of models. For *TLR4* and *TLR5*, all model comparisons (M1a vs. M2a, M7 vs. M8, and M8a vs. M8) significantly favored the positive selection model compared with the neutral model (*TLR4*, *P *≤* *0.017; *TLR5*, *P *≤* *7.2 × 10^−23^ for all comparisons), indicating that *P. papua TLR4* and *TLR5* have likely undergone positive selection. In contrast, for *TLR7*, the data were not a significantly better fit to the positive selection model compared with the neutral model (*P *=* *1), therefore, the null hypothesis of codons being negatively and neutrally selected was not rejected.

For *TLR4* and *TLR5*, we then used the Bayes Empirical Bayes (BEB) algorithm to infer the posterior probability that a particular codon has experienced positive selection. For *TLR4*, three codons were predicted to have undergone positive selection at posterior probability of >0.90 under model M2a (supplementary table S6*A–C*, Supplementary Material online). All but one of the *TLR4* polymorphic residues (12, 82, 236, 316, and 445) were located in the extracellular (leucine-rich repeat, LRR) domain, of which two were positively selected (12 and 236). The final positively selected site (659) was located in the transmembrane domain (fig. 6). Of the three selected sites, one (12 V/A) is a relatively nonconservative change (see supplementary table S7, Supplementary Material online, for amino acid distance metrics). One site in TLR4 (12) has previously been found to be under positive selection in birds (Velová et al. 2018), whereas the remaining two (236 and 659) are novel selected sites in penguins (supplementary table S8, Supplementary Material online).

For TLR5, there were 13 amino acid variants, of which nine were predicted to have undergone positive selection at posterior probability of >0.90 in both M2a and M8 (supplementary table S6*D–F*, Supplementary Material online). Of these, four were located in the extracellular domain (10, 285, 442, and 535), one was in the transmembrane domain (667), and four were located in the TIR (intracellular) domain (698, 747, 788, and 845; fig. 6). Two sites (442 and 698) have previously been reported as being positively selected in other birds (Grueber et al. 2014; Velová et al. 2018), whereas the remaining seven sites are novel selected sites in penguins. Interestingly, one site in the TLR5 extracellular domain (285) is adjacent to two residues known to be important for flagellin binding in Interface B (Yoon et al. 2012; Song et al. 2017), and so could be important for ligand preference (supplementary table S8 and fig. S2, Supplementary Material online). Four positively selected sites in TLR5 were nonconservative changes (10C/Y, 442A/V, 667S/F, and 788S/C). It is interesting to note that the nonconservative TLR5 667F/S polymorphism is located in the transmembrane domain, a region that is typically constrained by the physicochemical requirement to embed in the cell membrane. Furthermore, the homology-based methods of amino acid substitution consequence prediction SIFT (Ng and Henikoff 2003) and PolyPhen-2 (Adzhubei et al. 2013) both predict the S667F change to be of high functional consequence (SIFT score = 0.01; PolyPhen-2 score = 0.998; supplementary table S9, Supplementary Material online). Transmembrane integrity is predicted to remain intact, despite the nonconservative polymorphism, and the Phobius tool (Käll et al. 2004) predicts the transmembrane domain is unchanged by the polymorphism. Although the vast majority (57/64, 89% of available sequences) of avian TLR5 has a serine in this position in the transmembrane domain, only one other bird—the Northern Fulmar (*Fulmarus glacialis*)—has a phenylalanine in this position (supplementary fig. S3, Supplementary Material online), indicating there likely to have been a functional consequence to the S667F change in the Gentoo penguin.

### Functional Analysis of Selected TLR5 Residues

Although in silico methods can be useful indicators of protein residues under selection, functional study is the only means to isolate the selected phenotype and its relevance. In order to assess whether key selected sites identified in the positive selection analyses have functional consequences, we developed an in vitro assay using transient expression of TLRs in a reporter cell line.

**Fig. 7.**
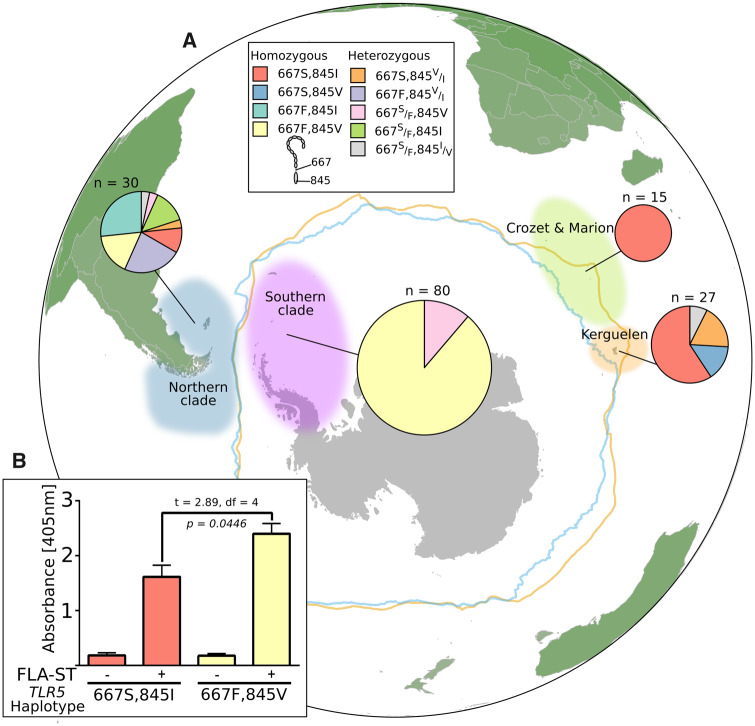
Distribution and functional differences of two selected amino acid residues in TLR5. (*A*) Mapped areas represent the four clades of *Pygoscelis papua* as determined by HVR1 analysis. Pie charts represent the allele proportions at the two selected residues in TLR5, and chart area is proportional to the number of samples. (*B*) Bar chart displays NF-kB response of the two genotypes following stimulation with FLA-ST.

Since extracellular domain polymorphisms in TLRs are likely to give rise to preferences in ligand type (Faber et al. 2018), we focused on TMD/TIR domain polymorphisms that are likely to give rise to differential signaling in response to the same agonist. Given that five *TLR5* polymorphisms were located in the TMD/TIR domain, we tested the two polymorphisms with the highest posterior probability of selection from the PAML analysis—residues 667 (TMD) and 845 (TIR). Polymorphisms at these positions segregate well with geographical location: birds from Crozet and Marion were all homozygous for the 667S/845I haplotype, whereas 87.3% (*n* = 71) of birds from colonies South of the Polar Front were homozygous for the derived haplotype, 667F/845V (fig. 7). In the Kerguelen Islands, the ancestral 667S/845I haplotype predominated, but variants at both positions were present at lower frequencies. South American/Falklands/Malvinas colonies were the most diverse at the positions of interest, where 46.7% (*n* = 14) of birds were heterozygous at one or both of the sites. Overall, however, polymorphisms at these loci tended to cosegregate: 72.4% (*n* = 110) of birds were homozygote for either the ancestral (667S/845I) or derived (667F/845V) haplotypes.

Given the strong tendency for the alleles at these positions to cosegregate at the extremes of the range of *P. papua*, and the fact that these were the TIR/TMD polymorphisms with the highest likelihood of positive selection, the functional consequences of altering both residues together were investigated. FLAG-tagged constructs of both of the TLR5 TIR/TMD variants with the same LRR domain were transiently expressed in *TLR5*^*−*^^*/*^^*−*^ HEK-Blue Null1 NF-κB reporter cells and protein expression levels were normalized using an anti-FLAG ELISA. Cells expressing either constructs were then treated with *Salmonella enterica* serovar Typhimurium-derived flagellin, or PBS control, and the NF-κB response was measured. Cells expressing either construct responded to flagellin, demonstrating that the *P. papua* TIR domain interacts efficiently with human adapter molecules. Interestingly, there was a marked enhancement (∼1.5-fold, *P *=* *0.04) of the flagellin response in the variant that was predominantly found in the Southern Gentoo penguin clade compared with the variant found in the Indian Ocean clades, suggesting that the derived haplotype 667F/845V has enhanced signaling capability compared with the ancestral genotype 667S/845I (fig. 7). These data provide further evidence that *P. papua TLR5* has undergone positive selection for different immune capabilities during the expansion of the species below the Polar Front toward the Antarctic Peninsula.

## Discussion

All vertebrates are subject to challenge by a plethora of pathogens that can exert strong selective pressures on host populations. The innate immune system, and in particular the TLRs, is responsible for both recognizing, and responding to, a pathogen threat by inducing inflammation and priming the adaptive immune response. To investigate TLR adaptation in Gentoo penguins, we sequenced the entire coding regions of *TLR4, TLR5*, and *TLR7*, which recognize products from bacterial and viral pathogens. Multiple individuals were sequenced from colonies at the extremes of the species’ range (∼8,000 km between the most distant colonies), providing an extensive geospatial component to our analysis. We found spatially associated patterns of diversity in the TLRs, although greater diversity was observed in *TLR4* and *TLR5* compared with *TLR7*. Furthermore, clear evidence of positive selection in both *TLR4* and *TLR5* was identified, which was further reinforced by the demonstration that two of the TLR5 TMD/TIR domain polymorphisms are sufficient to alter the magnitude of responsiveness to flagellin. To our knowledge, no other TLR study outside of humans has supported predictions of positive selection with confirmation of functional polymorphism.

### Patterns of Diversification and Selection

Most studies of TLR genetic diversity in wild populations have investigated small, bottlenecked, and/or endangered avian populations (Grueber et al. 2012, 2013; Hartmann et al. 2014; González-Quevedo et al. 2015; Dalton, Vermaak, Roelofse, et al. 2016; Dalton, Vermaak, Smit-Robinson, et al. 2016). In these populations, drift, rather than selection, is suspected to have been the main driver of sampled diversity due to recent bottlenecks or pronounced founder effects. Several studies have documented TLR diversity in domesticated animals such as pigs (Darfour-Oduro et al. 2016), cows (Novak et al. 2019), and chickens (Świderská et al. 2018), but large-scale studies on whole-gene TLR variation in a wild population are lacking. Although study design can dramatically affect the diversity detected in different studies, it is noteworthy that with Gentoo penguins *TLR5* exhibited higher diversity than *TLR4*, whereas with domestic chicken breeds (*n* = 110, 25 breeds; Świderská et al. 2018) and grey partridge (*n* = 10; Vinkler et al. 2015), *TLR4* was more diverse than *TLR5* (supplementary table S10, Supplementary Material online). The diversity of *TLR7* (compared with *TLR4* and/or *TLR5*) was relatively low in Gentoo penguins, domestic chicken breeds, and grey partridges (supplementary table S10, Supplementary Material online).

To provide an internal reference for TLR diversity, we sequenced the mitochondrial HVR1 as a neutral marker in the same individuals. In line with previous studies, we found evidence of at least four deeply divergent clades in *P. papua* based on HVR1 sequence (Vianna et al. 2017; Clucas et al. 2018; Pertierra et al. forthcoming). These more recent analyses support a revision, first proposed by de Dinechin et al. (2012), of the previously accepted two subspecies model that was based on morphological characteristics (Stonehouse 2008). These four clades are likely to have much greater divergence (millions of years) than what would be expected at the intraspecific level. We found evidence of differentiation according to this underlying population structure in *TLR4* and *TLR5* which further supports the argument for taxonomic revision of the species, with particular focus on the classification of colonies in South Georgia and the Indian Ocean. Conversely, *TLR7* was highly conserved across the species range and is clearly not subject to the same selection pressures as *TLR4* and *TLR5*. Overall, our study highlights that the genetic differentiation across Gentoo penguin clades is not just driven by drift but by clear population-specific adaptations to the environment.

Diversity has been widely reported to vary between different families of TLRs, particularly comparing extracellular and intracellular TLRs. Some authors have reported that TLRs that respond to viral ligands are more likely to be under purifying selection, at least in mammals (Barreiro et al. 2009; Wlasiuk and Nachman 2010; Wang et al. 2016; Kloch et al. 2018), although the pattern may not be consistent in birds, with *TLR3* displaying the greatest number of nonsynonymous variants of the four TLRs tested in different chicken breeds (Świderská et al. 2018), and *TLR7* diversity in the house finch (*Carpodacus mexicanus*) far exceeding that of *TLR4* and *TLR5* (Alcaide and Edwards 2011). Consistent with the pattern observed in mammals (and also the lesser kestrel, *Falco naumanni*; Alcaide and Edwards 2011), we observed overall nucleotide diversity measurements that were several times higher for the extracellular/bacterial TLRs *4* and *5* (*TLR4*: 6.1 × 10^−4^ ± 0.5 × 10^−4^; *TLR5*: 14.8 × 10^−4^ ± 0.6 × 10^−4^) compared with the intracellular/viral *TLR7* (1.0 × 10^−4^ ± 0.1 × 10^−4^), indicating strong purifying selection for maintenance of function in *TLR7*. In addition, we found no evidence of any codons under selection in *TLR7*, compared with three and nine sites in *TLR4* and *TLR5*, respectively, similar to the pattern of positively selected residues reported in several avian species (Alcaide and Edwards 2011).

Within TLR sequences, levels of variation are not uniformly distributed across the domains of the receptor. TLRs are type I integral membrane glycoproteins with highly conserved architecture across large phylogenetic distances (Botos et al. 2011). Typical TLR structure comprises an N-terminal extracellular (or intraluminal for intracellular TLRs) LRR domain for ligand binding, a single transmembrane helix, and a C-terminal cytoplasmic signaling (Toll/interleukin-1 receptor, TIR) domain interacting with intracellular adapter proteins (Bell et al. 2003). The LRR domain directly binds microbe-derived ligands in all known vertebrate TLRs, with the exception of TLR4 recognition of lipopolysaccharide via an accessory molecule, myeloid differentiation factor, MD-2 (Park et al. 2009). Since the LRR domain represents the interface between host and pathogen, and pathogens exhibit variable MAMPs to evade detection (Andersen-Nissen et al. 2005), there is often an excess of diversity in the LRR domain compared with the TIR domain (Świderská et al. 2018; Velová et al. 2018). In contrast, TIR domains interact with adapter proteins such as MyD88 (myeloid differentiation primary-response protein 88) which are shared between several TLRs, although a MyD88-independent pathway also facilitates TLR3 and TLR4 signaling (Akira and Takeda 2004). Unsurprisingly, TIR domains were found to be much more highly conserved than their corresponding extracellular domains in a study of 366 vertebrate TLRs from 96 species with the exception of *TLR10* (Mikami et al. 2012). Within species, the same trend is evident: in a study of *TLR3*, *4*, *5*, and *7* diversity across domestic chicken breeds, only 3 of the 46 nonsynonymous polymorphisms (two in *chTLR3* and one in *chTLR7*) were located in the TIR domain (Świderská et al. 2018).

In line with previous evidence for the asymmetric distribution of polymorphisms in TLR domains, we identified an excess of polymorphisms in the LRR domain compared with the TIR domain in two of the three TLRs studied (*TLR4*: 8 LRR vs. 0 TIR; *TLR7*: 6 LRR vs. 0 TIR). Interestingly, *P. papua TLR5* contained a greater number of TIR domain polymorphisms than would be expected from other species (11 LRR vs. 7 TIR), particularly given the LRR is over three times the length of the TIR domain. Of the seven *TLR5* TIR domain polymorphisms, five were nonsynonymous substitutions, suggesting that the TIR domain of *TLR5* has been under selection to modulate signaling intensity.

Somewhat surprisingly, we also identified nonsynonymous polymorphic sites in the transmembrane domains of both TLR4 (659 Ala/Thr) and TLR5 (667 Ser/Phe). The transmembrane domain is an uncommon location for TLR polymorphisms, presumably because the region is highly constrained by chemical and functional requirements. As such, the effects of polymorphisms in this region are often large. For instance, the human TLR1 602S variant is associated with disrupted cell surface localization of the receptor but is protective against pathology associated with leprosy. It is also noteworthy that the Gentoo penguin TLR5 transmembrane polymorphic site (667) identified in this study is highly conserved elsewhere in avian phylogeny. Of the other birds with published TLR5 sequences, displayed in the alignment (supplementary fig. S3, Supplementary Material online), 57 (89%) have a serine (ancestral *P. papua* genotype) at this position in the transmembrane domain, and only one other bird—the Northern Fulmar (*F. glacialis*)—has a phenylalanine residue (derived *P. papua* genotype). The high conservation of serine at this position in the protein points to a widespread pressure for maintenance of function across avian phylogeny, and provides more evidence of a positively selected residue with functional consequences.

### Functional Polymorphisms in *TLR5* Support Positive Selection

We identified a number of positively selected codons in both *TLR4* and *TLR5*, making both of these receptors candidates for further functional investigation. Polymorphisms in TLR LRR domains have the potential to yield preferences for subtly different microbial ligands, such as LPS or flagellins from different bacterial species (Nahori et al. 2005; Faber et al. 2018). However, very limited data are available regarding which pathogens are present in the environments of each of the Gentoo penguin clades, and therefore elucidation of any differences in ligand preference will require further study. We did, identify one positively selected site in TLR5 (285) that is adjacent to two important resides for flagellin binding in Interface B (Yoon et al. 2012; Song et al. 2017). This site would be a good candidate for functional investigation of changes in flagellin ligand preferences, but this would be difficult in the absence of known flagellin variants in candidate *P. papua* pathogens. Instead, we chose to investigate TIR and transmembrane domain polymorphisms for functional consequences because these can yield signaling intensity differences in response to the same agonist (Faber et al. 2018). Given that the TIR domain of TLR4 did not show any nonsynonymous polymorphisms, we focused on the TLR5 TIR/transmembrane domain, and in particular the two residues with the strongest signature of positive selection (667 and 845). Site 667 was likely to be of significant functional consequences because of its transmembrane location, nonconservative amino acid change (serine to phenylalanine) and both SIFT and PolyPhen-2 predicting the change to be of high importance.

The Gentoo penguin is reported to have undergone a circumpolar expansion, with ancestral populations in the Indian Ocean seeding northern populations that expanded into the Atlantic, and further expansions south of the Polar Front and to the West Antarctic Peninsula—the southernmost extreme of the range (de Dinechin et al. 2012; Peña et al. 2014). It is interesting to note that one of the ancestral Indian Ocean clades (Marion and Crozet archipelagos) is completely dominated by birds of the 667S/845I genotype, whereas the most derived clade of Gentoo penguins south of the Polar Front are almost entirely dominated by the 667F/845V genotype. These data may reflect an incipient selective sweep of the 667F/845V genotype in Southern Gentoo penguins.

An alternative explanation for the reduction in diversity at residues 667 and 845 could be genetic bottlenecking during the expansion of *P. papua* south of the Polar Front. However, evidence from neutral markers in this study and a previous study (Levy et al. 2016) reveals that neutral variation is maintained in the Southern Gentoo penguin colonies at levels comparable with the northern Atlantic Gentoo penguin clade up to the southernmost extreme of the range. In *TLR5*, we also saw no correlation between census population size and haplotype diversity. These findings support the hypothesis that a selective sweep, rather than a bottlenecking event, is responsible for the near-fixation of the TLR5 667F/845V haplotype in the Southern Gentoo penguin. Perhaps more importantly, the finding that the 667F/845V haplotype has enhanced signaling capability provides a functional basis for selection of this TLR5 haplotype.

### Potential Drivers of Selection

Toll-like receptors, like other genes of the immune system, are subject to competing types of selection. Balancing selection works to maintain diversity at a population level in response to the diversity of pathogens in the environment, as was recently proposed in TLRs of the bank vole (*Myodes glareolus*; Kloch et al. 2018). In contrast, purifying selection may predominate (to retain key functionality), which has been described in large-scale studies of human TLRs in different ethnic backgrounds (Mukherjee et al. 2014). Finally, positive selection may promote the fixation of novel variants that confer a fitness advantage in the response to pathogens. In the present study, we found evidence of positive selection in *TLR4* and *TLR5*, which likely indicates that the pathogen composition differs substantially between distinct locations in the Gentoo penguin’s range.

Spatial heterogeneity in the profile of pathogens that afflict Gentoo penguins would be a key driver for the patterns of selection identified in the TLR variants. Latitudinal species diversity gradients have been described for pathogens (and their hosts) (Rohde and Heap 1998; Dionne et al. 2007; Guégan et al. 2007), which might suggest fewer pathogens in Antarctic species. However, a diverse range of pathogens is found in these environments (discussed below). Moreover, within the Gentoo penguin's range, there are diverse biotic and abiotic characters that exhibit spatial variation (Trathan et al. 2007; Barbosa and Palacios 2009; Barbosa et al. 2013; Chown et al. 2015; Carpenter-Kling et al. 2019) and these factors will affect the transmission of pathogens. Indeed, the regionalized selection of TLR alleles in different sectors of the Gentoo penguin's range supports the premise that different challenges are more prevalent or pathogenic in different populations.

The dense colonial conditions and ubiquitous guano (feces) that characterize Gentoo penguin habitats provide ideal conditions for the transmission of a wide range of pathogens transmitted by direct contact or feces. Furthermore, penguins as a group are known to be highly susceptible to a variety of infectious diseases, including, avian cholera (Jaeger et al. 2018), avian pox (Kane et al. 2012), avian malaria (Fix et al. 1988; Grilo et al. 2016), and aspergillosis (Flach et al. 1990). A number of infection associated mass mortality events have been documented in both wild and captive penguin populations (Grimaldi et al. 2015). However, little is known about the pathogens that exist in sub-Antarctic and Antarctic regions, their prevalence, or their fitness costs on penguin populations. Limited data are available on the prevalence of diseases in penguin populations (Clarke and Kerry 2000; Barbosa and Palacios 2009; Woods et al. 2009; Grimaldi et al. 2015), and most studies rely upon short notes, observations, and case reports closely tied to obvious signs of disease and mass mortality in well-studied and highly visited penguin colonies. Studies that survey the environmental and host microbiomes to characterize pathogen presence in polar regions remain limited to sites near major polar research stations, have small sample sizes, and/or do not cover large spatial and temporal ranges (Zdanowski et al. 2004; Dewar et al. 2013, 2014; Fan et al. 2013; Ma et al. 2013).

The presence of Gram-negative bacteria exhibiting both lipopolysaccharides and flagella, including *Campylobacter*, *Escherichia*, *Salmonella*, and others, has been demonstrated in Gentoo penguin colonies (Dimitrov et al. 2009; Bonnedahl 2011; Barbosa et al. 2013; González-Acuña et al. 2013; García-Peña et al. 2017). However, it is not known whether any of these (or other bacterial pathogens) vary across the Gentoo penguin’s range, or may have played a role in the selection of Gentoo penguin *TLR4* or *TLR5* variants.

Studies of single-stranded RNA viruses (which would typically be recognized by TLR7) are similarly lacking in Gentoo penguins. Though single-stranded RNA viruses, including the causative agents of Newcastle disease virus and avian influenza, have occasionally been detected in *Pygoscelis* penguins through immunological assays and direct isolation (Morgan and Westbury 1988; Wallensten et al. 2006; Neira et al. 2017; Olivares et al. 2019; Wille et al. 2019), the fitness consequences of viral infection on penguin populations are unknown. We know of only one case report from Signy Island, where evidence of a puffinosis outbreak (normally caused by a Coronavirus) was described in Gentoo penguin chicks (MacDonald and Conroy 1971). The viral drivers behind the strong purifying selection we observed in *TLR7* are unknown, but it could be that the ssRNA viruses that affect Gentoo penguins are less diverse across the species range than flagellated Gram-negative bacteria.

Sympatric interactions with a diversity of migratory flying birds and their parasites may be important contributors to pathogen diversity in Gentoo penguin colonies. Birds such as albatrosses, petrels, shearwaters, sheathbills, shags, gulls, terns, and skuas are often observed in close proximity to Gentoo penguin colonies and there are 46 species recognized in Antarctica alone (Lepage et al. 2014). There is some evidence that ectoparasites and blood parasites are transmitted between co-occurring bird species (Barbosa et al. 2011; Levin et al. 2013). It is plausible that cross-species transmission events are important in cross-colony transmission and structuring the profile of pathogens afflicting particular Gentoo penguins.

It remains unclear why the two functionally tested TMD/TIR residues in TLR5 would confer increased responsiveness to flagellin in Gentoo penguins south of the Polar Front. TLR signaling must be tightly controlled and aberrant TLR-induced inflammation can lead to immune pathology, toxic shock syndrome, and death. It is unsurprising, therefore, that TLR polymorphisms have been described that confer reduced sensitivity to their agonist and a state of tolerance. For instance, the replacement of a highly conserved proline residue by a histidine at position 712 of TLR4 confers endotoxin resistance in certain strains of mice (Qureshi et al. 1999). It could be that the exposure to (or diversity of) pathogens is decreased for the Southern Gentoo penguin clade compared with other clades of Gentoo penguins, and thus individuals can tolerate enhanced signaling to a prevailing infection. Alternatively, the enhanced signaling could be a manifestation of the Southern Gentoo penguin adapting to a particular pathogen that is present in the West Antarctic Peninsula and absent elsewhere. The finding of adaptive changes in the Gentoo penguin immune system necessitates a much better understanding of the pathogen threats faced by Gentoo penguins in order for their significance to be realized.

### Concluding Remarks

This wide-ranging immunogenetic study of TLRs in wild Gentoo penguins reveals differential selection and adaptation to local pathogen pressure. Although the drivers behind the observed patterns of diversity and selection remain unclear in the context of currently available data, it is clear that the Gentoo penguin has undergone adaptation to local pathogen assemblages across its range.

Infectious disease threats to penguins are likely to become ever more severe in the coming decades given the rapidly changing polar climate (Lynch et al. 2012; Mayewski 2012). There is also evidence of reverse zoonosis of enteric bacteria being transmitted from humans to sea bird species in Antarctica (Cerdà-Cuéllar et al. 2019), which could further increase transmission, especially in light of increasing tourist and scientific research program presence in Antarctica. Although the Gentoo penguin is not currently one of the 13 out of 18 penguin species with a conservation state of threatened or near-threatened, certain sub-Antarctic populations have experienced sharp declines (Crawford et al. 2003, 2009, 2014; Lescroel and Bost 2006). Consequently, the vulnerability of pathogen-naïve populations of penguins should not be underestimated, nor should the importance of the Gentoo penguin as a sentinel species in the Southern Ocean (Carpenter-Kling et al. 2019).

Our findings have important implications for the conservation of not just Gentoo penguins but also many other vertebrate species, both in the wild and in captivity. Until now, most efforts to genetically delineate conservation units have relied mostly on neutral markers. The ever-increasing availability of genomic data allows targeted analysis of pathogen-recognition and other immune genes to assess whether different populations possess specific functional adaptations to their environments and should therefore be conserved separately. The approach used here, together with pathogen discovery and surveillance systems, could better define conservation units in species that occupy varied habitats and ecological niches in order to focus resources on potentially susceptible populations.

## Materials and Methods

### Sample Collection

This study used 155 blood samples from Gentoo penguins, previously obtained in the framework of other projects. Samples were collected between 1999 and 2017 at the 14 sites shown in figure 1 (details in supplementary table S11, Supplementary Material online). To take blood, penguins were held with the flippers restrained and the head placed under the arm of the handler, or they were wrapped in cushioned material covering the head and preventing movement, to minimize stress during handling (Le Maho et al. 1992). A second handler took up to 1 ml blood from the brachial, intertarsal, or jugular vein using a 25- or 23-G needle and 1-ml syringe or capillary, after cleaning the area with an alcohol swab. Total restraint time was generally 2–3 min. The animal was then released at the edge of the colony and observed to ensure it returned to its normal behavior. Blood was stored in 95% ethanol or Queen’s lysis buffer at −20 °C for transport at room temperature and subsequent storage at −20 °C upon arrival. All blood samples were imported under the appropriate animal by-product import licenses.

Sampling was conducted under permits from each site’s territorial government or governing agency. These permits for animal handling were issued following independent institutional ethical review of the sampling protocols, in accordance with Scientific Committee for Antarctic Research (SCAR) guidelines.

### DNA Extraction

DNA for samples from MO, CB, BI, COP, and JP was extracted from blood samples using QIAGEN DNeasy Blood and Tissue kits. The digestion step was modified to include 40 μl proteinase K and extended to 3 h for blood samples. Details of the modifications made to the protocols for tissue samples are available in (Younger, Clucas, et al. 2015; Younger, Emmerson, et al. 2015). All these samples were treated with 1 μl Riboshredder (Epicentre) to reduce RNA contamination and DNA was visualized on a 1% agarose gel to confirm high-molecular weight DNA was present. DNA concentration and purity were measured on a Qubit and Nanodrop (Thermofisher Scientific), respectively. These samples are stored at the University of Oxford for future analysis. DNA from all other sampling sites was isolated using a modified salt protocol (Aljanabi and Martínez 1997), with details in Vianna et al. (2017), stored at the Pontificia Universidad Católica de Chile for future analysis.

### TLR Genotyping

Primers for *TLR4*, *5*, and *7* coding regions were designed using the primer design feature based on Primer3 2.3.7 (Untergasser et al. 2012) in Geneious v.11.0 (Biomatters, http://www.geneious.com). Reference coding sequences for primer design were derived from the congeneric Adélie penguin (*P. adeliae*) reference genome (GenBank accession JMFP01000000) and unpublished Gentoo penguin genomic data. *TLR4* amplifications for samples from MO, CB, BI, COP, and JP were conducted in 12 µl volumes (9 µl Qiagen *Taq* PCR Master Mix, 2 µl 10 µM forward and reverse primer mix, and 1 µl of template DNA diluted 1:100). *TLR5* and *TLR7* amplifications for these samples were conducted in 25 µl volumes containing 5 µl 5× Phusion High Fidelity (HF) Buffer (New England Biolabs, UK), 0.5 µl 10 mM dNTPs, 1.25 µl of 10 µM forward primer, 1.25 µl of 10 µM reverse primer, 2 µl of template DNA diluted 1:100, 0.25 µl of Phusion Hot Start Flex DNA Polymerase (New England Biolabs, UK), and 14.75 µl nuclease-free water. One GC-rich region in *TLR7* required the use of Phusion GC buffer, rather than HF buffer for amplification. PCR products were visualized on a 1% agarose gel stained with SYBR Safe. The primers and PCR reaction conditions are fully detailed in the Supplementary Material online (supplementary table S12, Supplementary Material online). PCR products were sequenced using Macrogen Europe’s EZ-Seq (*TLR4*) or Eco-Seq (*TLR5* and *TLR7*) services (http://www.macrogen.com, Netherlands) for purification and Sanger sequencing, using the same PCR primers for sequencing.

For all remaining sampling sites, which underwent whole-genome sequencing, a total of 100 ng of genomic DNA was fragmented to an average of 350 bp to construct paired-end libraries using the Illumina TruSeq Nano kit with the included indexed adapter and barcode. A total of six PCR cycles were used for enrichment, purified with sample purification beads, quantified using a Qubit fluorometer, and then sequenced to ∼20× coverage with 150 paired-end reads using an Illumina HiSeq X platform at MedGenome.

Sequences for each TLR coding region were assembled, edited, and aligned using Geneious v.11.0. For *TLR4*, which has multiple exons along the coding region, exon sequences were extracted and concatenated for further analysis. Heterozygous sites and single-nucleotide polymorphisms were detected by visually examining chromatograms. In cases of doubt, resequencing was accomplished so that only high-quality reads from multiple sequencing runs were called as single-nucleotide polymorphisms. All heterozygous sites also had homozygous individuals within the data set, and each gene had at least one haplotype homozygous across the full length of the gene. All alleles were verified using a combination of multiple independent Sanger sequencing runs and where available, the whole-genome sequencing data. The International Union of Pure and Applied Chemistry (IUPAC) code for degenerate nucleotides was used to label heterozygous positions.

### mtDNA Genotyping

For mitochondrial DNA, the hypervariable region of the mitochondrial control region (HVR1), also known as Domain I, was amplified using the primers GPPAIR3F and GPPAIR3R (Clucas et al. 2014) for samples from MO, CB, BI, COP, and JP. Amplifications were conducted in 25 µl volumes according to the manufacturer’s instructions, using Phusion Hot Start Flex DNA Polymerase (New England Biolabs, UK) and 2 µl of template DNA diluted 1:100. Amplifications involved a two-step PCR, with an initial cycle of 98 °C for 30 s, 40 cycles of 98 °C for 10 s and 72 °C for 20 s, followed by a 10-min extension at 72 °C. PCR products were visualized on a 1% agarose gel stained with SYBR Safe. PCR fragments were purified using an ethanol/sodium acetate precipitation, and sequencing was performed using the Applied Biosystems BigDye Terminator v3.1 sequencing kit (Applied Biosystems) with the same PCR primers as sequencing primers.

Published mtDNA HVR1 sequences for the samples from BI (GenBank accessions KJ646314–KJ646330, *n* = 16) and COP (KJ646361–KJ646382, *n* = 21) were included in the analysis for the relevant individuals. mtDNA data were not available for the individuals from two Antarctic sites. Though other individuals from those sites have sequence data available, we only included data from individuals sequenced for both TLRs and HVR1.

Individual mtDNA fragments from all remaining sites (CR, MAR, COU, BR, MT, SIG, SP) were amplified using the primers tRNAGlu and AH530 (Roeder et al. 2002). These reactions included 0.4 μM of each primer, 1.5 mM 1× of PCR reaction buffer, MgCl_2_, 200 μM of each dNTP, and 1 U of Taq Polymerase Platinum (Invitrogen) in a two-phase touchdown program (Korbie and Mattick 2008): 1) 10 min at 95 °C, and 11 cycles of 95 °C for 15 s; a touchdown with an annealing temperature of 60–50 °C for 30 s, with one cycle per 1 °C interval, and 72 °C for 45 s; 2) 35 amplification cycles at 95 °C for 15 s, 50 °C for 30 s, and 72 °C for 45 s; and a final extension period of 30 min at 72 °C. The purification of these PCR products and sequencing was carried out by Macrogen using an ABI PRISM 3730XL.

Only overlapping segments of the HVR1 sequences common to all samples were used by aligning and editing with Geneious v11.0. Consensus sequences for the resulting 273-bp region of interest were extracted for analysis.

### Population-Level Analyses

Haplotypes were inferred for each of the diploid TLR loci using the PHASE algorithm (Stephens et al. 2001) implemented in DnaSP 6.12.10 (Rozas et al. 2017) with 10,000 iterations and 1,000 burn-in iterations. Phased haplotype data were used as input to determine standard genetic diversity measures of each population, including number of polymorphic sites, haplotypes, haplotype diversity, and nucleotide diversity, using DnaSP 6.12.10 and Arlequin v3.5.1.3 (Excoffier and Lischer 2010). Minimum spanning haplotype networks were constructed and visualized using PopART 1.7 (Bandelt et al. 1999; Leigh and Bryant 2015) for each locus. DnaSP was also used to identify synonymous and nonsynonymous polymorphic sites and frequencies. Arlequin was used to calculate Tajima’s *D* (Tajima 1989), and Fu’s *F*s (Fu 1997). FSTAT v.2.9.3 (Goudet 1995) was used to calculate allelic richness, taking into account differences in sample size.

Because TLR nucleotide diversity has been observed to have a correlation to population size in some island bird populations (Gilroy et al. 2017), we evaluated the relationship between Gentoo penguin census population sizes (*N*_c_) and haplotype diversity (*H*_d_) at each locus. Gentoo penguins are philopatric but also show evidence of admixture within island groups and adjacent coastlines (Levy et al. 2016 and Vianna et al. 2017). For this reason, the census population sizes selected for the analysis were numbers of breeding pairs from the most recent available surveys of each archipelago or region (in the case of the South Shetland Islands and Western Antarctic Peninsula). The *N*_c_ size and source survey reference are available in supplementary table S3, Supplementary Material online. Spearman’s rank correlations and *P* values were calculated for each diversity-size comparison.

For population differentiation comparisons, Arlequin was used to calculate pairwise *F*_ST_ distances based on haplotype frequencies (Weir and Cockerham 1984), and pairwise *Φ*_ST_s for the TLR and mtDNA sequences (Excoffier et al. 1992). FindModel (Posada and Crandall 1998) was used to find the best fit substitution model for use in Arlequin. *Φ*_ST_ calculations for the TLR loci were obtained using the Tamura and Nei (1993) substitution model, whereas mtDNA *Φ*_ST_ analysis was carried out using the Kimura 2-Parameter model (Kimura 1980) with a gamma of 0.27. AMOVA was used to compute hierarchical F-statistics, with 10,000 permutations, to evaluate likely patterns of genetic structure, seeking to identify the population grouping that maximized the among-group variation (*F*_CT_) and minimized the variation among colonies within groups (*F*_SC_) (Excoffier et al. 1992). Significance of overall and pairwise genetic distances was computed using 1,000,000 permutations. We used the SGoF+ method (Carvajal-Rodríguez and de Uña-Alvarez 2011) within the Myriads software (Carvajal-Rodríguez 2018) to correct for multiple tests.

To test for isolation-by-distance, shortest distances by sea (during summer ice extent), in km, were computed between each sampling location, using Google Earth v7.3.2.5776 (supplementary table S5*B*, Supplementary Material online), which were then related to pairwise *F*_ST_ in a Mantel test, implemented in Arlequin.

### Population Divergence and Phylogeography

Phylogenetic reconstruction and estimates of divergence time were carried out using BEAST 2.5.2 (Bouckaert et al. 2019). The evolutionary model for mtDNA analysis was selected using jModelTest v. 2.1.10 (Darriba et al. 2012), testing 88 candidate models and selecting the best fit using the Bayes Information Criterion. All 88 models were within the 100% confidence interval, with HKY+G selected for further divergence analyses (Hasegawa et al. 1985). A total of 15 Adélie penguin (*P. adeliae*) and 15 Chinstrap penguin (*P. antarcticus*) mitochondrial HVR1 GenBank sequences (Clucas et al. 2014), aligned and cropped to the equivalent size of the Gentoo sequences to avoid bias, were included in the analysis as outgroups.

The most recent common ancestor prior was set for the *Pygoscelis* genus at 7.6 Ma (Subramanian et al. 2013), derived from the fossil calibration for *P. grandis* (Walsh and Suarez 2006), with a normal distribution, and SD (σ) of 1.3 Ma. A strict molecular clock with a starting prior of 1.0 and a Yule speciation process for branching rates, with uniform priors for birth and clock rates of 1.0 was applied. Four independent runs of 30 million MCMC chains were performed, logging parameters every 3,000 steps. The four independent runs were then combined using LogCombiner v.2.5.2 and assessed for convergence within Tracer v.1.7.1 (Rambaut et al. 2018). All parameters converged with ESS values >6,000. A maximum clade credibility tree was then generated using TreeAnnotator v.2.5.2 (part of the BEAST software distribution) and visualized in FigTree v.1.4.3.

### Selection Analyses

Phylogenetic inference was carried out on phased sequence data for each TLR locus using RAxML-NG using a GTR substitution matrix (Kozlov et al. 2019). To detect selection, maximum likelihood analysis of ratios of nonsynonymous to synonymous nucleotide substitutions (d*N*/d*S*; *ω*) was performed with the *codeml* package of programs in PAML v. 4.9 (Yang 1997, 2007). Various models were fitted to the multiple alignments: M1a (neutral model; two site classes: 0 < *ω*_0_ < 1 and *ω*_1_ = 1); M2a (positive selection; three site classes: 0 < *ω*_0_ < 1, *ω*_1_ = 1, and *ω*_2_ > 1); M7 (neutral model; values of *ω* fit to a beta distribution where *ω*  >  1 disallowed); M8 (positive selection; similar to M7 but with an additional codon class of *ω*  >  1); and M8a (neutral model; similar to M8 but with a fixed codon class at *ω*  =  1). Likelihood ratio tests were performed on pairs of models to assess whether models allowing positively selected codons gave a significantly better fit to the data than neutral models (model comparisons were M1a vs. M2a, M7 vs. M8, and M8a vs. M8). In situations where the null hypothesis of neutral codon evolution could be rejected (*P* < 0.05), the posterior probability of codons under selection in M2a and M8 was inferred using the BEB algorithm (Yang et al. 2005).

### In Silico Prediction of Polymorphism Functional Consequences

Physicochemical distances between amino acid variants were assessed using distance matrices provided by several authors (Sneath 1966; Epstein 1967; Grantham 1974; Miyata et al. 1979; Urbina et al. 2006; supplementary table S7, Supplementary Material online). Predicted functional consequences of amino acid substitutions were assessed using the homology-based tools SIFT (Ng and Henikoff 2003) and PolyPhen-2 (Adzhubei et al. 2013), using online servers (https://sift.bii.a-star.edu.sg/ and http://genetics.bwh.harvard.edu/pph2/; both accessed December 2019). Transmembrane domain positions were predicted using Phobius (Käll et al. 2004; http://phobius.sbc.su.se/; accessed December 2019).

### Functional Analysis of TLR5 Genotype Expression in CRISPR-Cas9 Edited HEK-Blue Cells

In order to functionally assess positively selected *TLR5* polymorphisms in vitro, two full-length *TLR5* sequences were synthesized including the two signaling domain polymorphisms that had the strongest signature of selection (GBlocks, IDT). Synthetic genes were cloned using the Gibson assembly method (Gibson et al. 2009) into the p3XFLAG-CMV-14 expression vector (Sigma) which incorporates a 3×-FLAG sequence on the C-terminus of the expressed construct. Insert-containing vector was purified using the ZymoPURE II plasmid Maxiprep with the optional endotoxin-removal step (Zymo). Both constructs were transiently expressed using TransIT-2020 (Mirus Bio) in custom HEK-Blue Null1 cells (InvivoGen) that had undergone genome editing using the CRISPR-Cas9 technique to disrupt endogenous human *TLR5*. Cells expressing Gentoo penguin *TLR5* constructs were challenged with *Salmonella* Typhimurium-derived flagellin (FLA-ST; InvivoGen) at 100 ng/ml and incubated for 24 h. Cell supernatants were harvested and NF-κB activity was assessed by measuring the absorbance at 405 nm on a FLUOstar Omega microplate reader (BMG Labtech) following the addition of p-nitrophenyl phosphate substrate, according to the manufacturer’s instructions (SIGMAFAST, Sigma). Expression levels were monitored by subjecting cell lysates to a direct anti-FLAG ELISA. Cell lysates were harvested in ice-cold RIPA buffer (ThermoFisher) and proteins were immobilized on high-bind ELISA plates (VWR) using coating buffer (BioLegend) overnight at 4 °C. Wells were blocked using StartingBlock (PBS) blocking buffer (ThermoFisher) for 1 h and then incubated with mouse monoclonal anti-FLAG M2 antibody (Sigma, 1:1,000) at 37 °C for 1 h, followed by incubation with goat anti-mouse IgG-HRP (ThermoFisher, 1:10,000) for 1 h at 37 °C. Reactive protein amount was then assessed by the addition of 3,3′,5,5′-tetramethylbenzidine substrate and measurement of absorbance at 650 nm. Expression data were then used to normalize signaling data. Statistical differences between means were determined by a two-tailed Student’s *t*-test, and statistical significance was considered to be *P *<* *0.05. Transfections were carried out in three independent wells per condition, and the experiment was conducted on at least three independent occasions. 

## Supplementary Material

msaa040_Supplementary_Information

## Notes

All sequence data generated in this study were deposited in GenBank under accession numbers MN394222–MN394376 (*TLR4*), MN313018–MN313169 (*TLR5*), MN312870–MN313017 (*TLR7*), and MN566362–MN566421 (mitochondrial control region, HVR1).
