# Supplementary material for: Evidence of Pathogen-Induced Immunogenetic Selection across the Large Geographic Range of a Wild Seabird
Source: Mol Biol Evol. 2020 Feb 25;37(6):1708–26. doi: 10.1093/molbev/msaa040 (PMC7253215; doi:10.1093/molbev/msaa040)
Supplement: msaa040_Supplementary_Information [file msaa040_supplementary_information.pdf]

Evidence of pathogen-induced immunogenetic selection across the large geographic range of a wild seabird

Supplementary Information

Supplementary Table S1. Polymorphisms and diversity indices for fourteen colonies of Gentoo penguin at three toll-like receptor loci and the mitochondrial hypervariable control region. (CR = Crozet Island; MAR = Marion Island; COU = Courbet Peninsula, Kerguelen; MO = Pointe du Morne, Kerguelen; CB = Cow Bay, Falkland/Malvinas Islands; BR = Bull Roads, Falkland/Malvinas Islands; BI = Bird Island, South Georgia; MT = Martillo Island, Tierra del Fuego; SIG = Signy Island, South Orkney Islands; COP = Copacabana (Admiralty Bay), King George Island, South Shetland Islands; SP = Stranger Point, King George Island, South Shetland Islands; BO = Bernardo O'Higgins Base, Western Antarctic Peninsula; GGV = Gabriel González Videla Base, Western Antarctic Peninsula; JP = Jougla Point, Western Antarctic Peninsula).

Locus	Colony (*N*)	*S*	*H*	H_d (SD)	π (SD) ($X10^{-4}$)	UPV	AR	*D*	*Fs*
TLR4	CR (7)	5	4	0.6593 (0.1203)	5.86 (1.9)	3	3.802	-0.17122	0.48000
	MAR (8)	5	4	0.7667 (0.0573)	9.80 (1.2)	4	3.858	2.17212*	2.06630
	COU (7)	8	7	0.6923 (0.1366)	8.32 (2.4)	4	5.286	-0.59171	-1.89341
	MO (15)	9	14	0.9034 (0.0362)	11.01 (0.8)	8	7.033	0.73200	-5.86857*
	CB (19)	2	2	0.0526 (0.0492)	0.41 (0.4)	1	1.263	-1.49106*	-0.61060
	BR (7)	0	1	0	0	1	1.000	0	0
	BI (18)	0	1	0	0	1	1.000	0	0
	MT (5)	0	1	0	0	1	1.000	0	0
	SIG (5)	4	3	0.5111 (0.1643)	6.01 (2.3)	1	3.000	0.32418	1.35813
	COP (22)	5	5	0.6342 (0.0511)	7.46 (0.7)	1	3.246	1.63019	1.71487
	SP (5)	4	3	0.5111 (0.1643)	6.45 (2.1)	1	3.000	0.62589	1.51568
	BO (6)	5	3	0.5455 (0.1436)	5.94 (2.3)	2	2.970	-0.31291	1.59874
	GGV (7)	4	2	0.2637 (0.1360)	4.14 (2.1)	1	1.934	-0.53129	2.69666
	JP (24)	4	3	0.0824 (0.0541)	1.12 (0.7)	1	1.417	-1.56204*	-0.73409
	Total (155)	13	21	0.478 (0.035)	6.1 (0.5)	9	3.446	-0.58350	-10.018
TLR5	CR (7)	1	2	0.4396 (0.1120)	1.66 (0.4)	2	1.999	0.84228	0.94377
	MAR (8)	1	2	0.1250 (0.1064)	0.47 (0.4)	2	1.625	-1.16221	-0.70010
	COU (7)	15	8	0.8571 (0.0774)	16.09 (2.9)	7	6.275	-0.40117	-0.88654
	MO (20)	13	17	0.8731 (0.0415)	12.30 (1.3)	14	6.697	0.19907	-6.77889*
	CB (18)	11	17	0.9159 (0.0309)	17.10 (0.7)	12	7.380	2.17976	-4.88768
	BR (7)	9	6	0.7692 (0.0895)	16.09 (1.9)	5	4.856	1.92919	1.04846
	BI (12)	5	5	0.4928 (0.1165)	5.25 (1.6)	4	2.883	0.10549	-0.03412
	MT (5)	9	6	0.8444 (0.1029)	15.72 (2.9)	5	6.000	1.34084	-0.04600
	SIG (5)	5	3	0.5111 (0.1643)	4.37 (2.3)	3	3.000	-1.38818	0.76172
	COP (21)	6	6	0.6527 (0.0485)	4.54 (1.0)	6	3.462	-0.36633	-0.68391
	SP (5)	1	2	0.2000 (0.1541)	0.76 (0.6)	2	2.000	-1.11173	-0.33931
	BO (6)	5	3	0.3182 (0.1637)	3.15 (2.1)	3	2.667	-1.83094*	0.32487
	GGV (7)	2	3	0.3846 (0.1494)	1.54 (0.6)	3	2.648	-0.95919	-0.85452
	JP (24)	1	2	0.1197 (0.0612)	0.45 (0.2)	2	1.512	-0.63670	-0.38233
	Total (152)	20	46	0.781 (0.022)	14.8 (0.6)	32	5.600	0.59898	-23.918

TLR7	CR (7)	0	1	0	0	1	1.000	0	0
	MAR (8)	0	1	0	0	1	1.000	0	0
	COU (7)	3	4	0.4945 (0.1506)	2.32 (0.8)	4	2.978	-0.70770	-1.11799
	MO (18)	2	3	0.3381 (0.0919)	1.13 (0.3)	2	2.275	-0.51702	-0.49065
	CB (17)	1	2	0.0588 (0.0546)	0.19 (0.2)	2	1.235	-1.13783	-1.31506
	BR (7)	1	2	0.3626 (0.1302)	1.16 (0.4)	2	1.945	0.32440	0.64281
	BI (13)	0	1	0	0	1	1.000	0	0
	MT (4)	1	2	0.4286 (0.1687)	1.37 (0.5)	2	2.000	0.33350	0.53626
	SIG (5)	2	3	0.6000 (0.1305)	2.13 (0.6)	2	2.800	-0.18393	-0.27178
	COP (21)	3	4	0.3751 (0.0902)	1.30 (0.3)	3	2.413	-0.90151	-1.43643
	SP (5)	1	2	0.4667 (0.1318)	1.49 (0.4)	1	2.000	0.81980	0.81801
	BO (6)	2	3	0.4394 (0.1581)	1.50 (0.6)	2	2.576	-0.84971	-0.72455
	GGV (7)	2	3	0.3846 (0.1494)	1.30 (0.5)	2	2.407	-0.95919	-0.85452
	JP (23)	4	5	0.3826 (0.0881)	1.34 (0.3)	4	2.510	-1.25133	-2.59143
	Total (148)	9	10	0.296 (0.034)	1.0 (0.1)	8	2.140	-1.67798	-9.379
**HVR1**	CR (7)	8	4	0.8570 (0.102)	123.5 (33.0)	-	-	0.10944	0.87029
	MAR (8)	5	4	0.7860 (0.113)	68.8 (20.5)	-	-	-0.16751	0.00547
	COU (2)	0	1	0	0	-	-	0	0
	MO (20)	28	16	0.9580 (0.033)	173.2 (10.0)	-	-	-0.93073	-7.01213*
	CB (16)	12	10	0.9000 (0.062)	116.4 (15.4)	-	-	-0.50145	-3.57558
	BR (7)	5	4	0.8570 (0.102)	77.6 (18.5)	-	-	0.13210	-0.04242
	BI (17)	9	8	0.8820 (0.047)	78.2 (13.8)	-	-	-0.75716	-3.70741*
	MT (5)	4	2	0.6000 (0.175)	88.9 (26.0)	-	-	1.64070	3.02249
	SIG (5)	6	5	1.0000 (0.126)	103.7 (18.8)	-	-	-0.19092	-2.37061*
	COP (21)	22	15	0.9670 (0.024)	151.4 (18.9)	-	-	-1.27487	-6.15999*
	SP (6)	16	6	1.0000 (0.096)	227.2 (34.2)	-	-	-0.77134	-1.77289
	JP (24)	18	15	0.9420 (0.031)	127.2 (18.6)	-	-	-1.03686	-7.42519*
	Total (138)	90	79	0.986 (0.003)	744.3 (56.6)	-	-	0.53097	-22.196

S = number of polymorphic sites
H = number of haplotypes
H_d = haplotype diversity (standard deviation)
π = nucleotide diversity
UPV = unique protein variants of translated haplotypes
AR = allelic richness, adjusted for sample size differences
D = Tajima's D test statistic, with * indicating $p < 0.05$
Fs = Fu's Fs test statistic, with * indicating $p < 0.02$

<u>Supplementary Table S2</u>. Pairwise F_{ST} values (Weir and Cockerham 1984) derived from haplotype frequencies (below diagonal), and pairwise Φ_{ST} values of genetic distance (above the diagonal), calculated using the Tamura and Nei substitution model (Tamura and Nei 1993), with associated p-values, among Gentoo penguin breeding sites based on toll-like receptor sequence data (tables S2A-C). mtDNA hypervariable region I Φ_{ST} values were calculated using the Kimura 2-Parameter (K80) substitution model (Kimura 1980) with a gamma of 0.27 (table S2D). Comparisons that are significant <0.01 after correction for multiple tests using (SGOF+) are shown in bold (Carvajal-Rodriguez and de Uña-Alvarez 2011).

S2A. *TLR4*

**Colony**	**CR**	**MAR**	**COU**	**MO**	**CB**	**BR**	**BI**	**MT**	**SIG**	**COP**	**SP**	**BO**	**GGV**	**JP**
**Crozet Island (CR)**	*	0.04120 (0.17969)	0.43041 **(0.00079)**	0.16276 (0.01178)	0.84905 **(0.00000)**	0.78209 **(0.00010)**	0.87027 **(0.00000)**	0.75072 **(0.00000)**	0.54200 **(0.00010)**	0.40124 **(0.00000)**	0.48925 **(0.00109)**	0.55826 **(0.00000)**	0.60966 **(0.00010)**	0.82233 **(0.00000)**
**Marion Island (MAR)**	0.00110 (0.40541)	*	0.20483 (0.01475)	0.0153 (0.24453)	0.66826 **(0.00000)**	0.54644 **(0.00000)**	0.69000 **(0.00000)**	0.50219 **(0.00158)**	0.31652 (0.01049)	0.25797 **(0.00400)**	0.26514 (0.01416)	0.33837 **(0.00446)**	0.38119 **(0.00228)**	0.65266 **(0.00000)**
**Courbet Peninsula (COU)**	0.24738 **(0.00158)**	0.13006 (0.14950)	*	0.04851 (0.10870)	0.21641 **(0.00485)**	0.12661 (0.04010)	0.25919 **(0.00030)**	0.08874 (0.10603)	0.00000 (0.41392)	0.06705 (0.08405)	0.00000 (0.50153)	0.00000 (0.39570)	0.00000 (0.35739)	0.18573 (0.00911)
**Pointe du Morne (MO)**	0.08383 (0.01109)	0.01953 (0.18993)	0.03074 (0.10365)	*	0.39773 **(0.00000)**	0.30278 **(0.00050)**	0.41555 **(0.00000)**	0.27201 **(0.00307)**	0.13365 (0.02901)	0.10606 **(0.00505)**	0.08702 (0.06831)	0.15195 (0.01366)	0.17420 (0.00871)	0.39423 **(0.00000)**
**Cow Bay (CB)**	0.71152 **(0.00000)**	0.58202 **(0.00000)**	0.28168 **(0.00069)**	0.38276 **(0.00000)**	*	0.00000 (0.99990)	0.00000 (0.99990)	0.00000 (0.99990)	0.30157 (0.01139)	0.31274 **(0.00010)**	0.34009 (0.01257)	0.18803 (0.01119)	0.12601 (0.07079)	0.00000 (0.61390)
**Bull Roads (BR)**	0.61538 **(0.00000)**	0.46925 **(0.00000)**	0.19231 (0.01554)	0.29735 **(0.00000)**	0.00000 (0.99990)	*	0.00000 (0.99990)	0.00000 (0.99990)	0.19940 (0.06257)	0.24712 **(0.00455)**	0.23125 (0.05425)	0.10916 (0.03168)	0.07692 (0.47787)	0.00000 (0.99990)
**Bird Island (BI)**	0.75224 **(0.00000)**	0.6231 **(0.00000)**	0.34289 **(0.00010)**	0.40973 **(0.00000)**	0.00000 (0.99990)	0.00000 (0.99990)	*	0.00000 (0.99990)	0.37725 **(0.00743)**	0.33222 **(0.00000)**	0.41492 (0.00812)	0.24677 **(0.00317)**	0.19101 (0.07247)	0.00997 (0.50589)
**Martillo Island (MT)**	0.57055 **(0.00020)**	0.42329 **(0.00020)**	0.14980 (0.04990)	0.26665 **(0.00010)**	0.00000 (0.99990)	0.00000 (0.99990)	0.00000 (0.99990)	*	0.14818 (0.21107)	0.22308 (0.01901)	0.17778 (0.20889)	0.06971 (0.32304)	0.04328 (0.49352)	0.00000 (0.99990)
**Signy Island (SIG)**	0.34300 **(0.00050)**	0.21259 (0.00772)	0.00000 (0.42926)	0.10256 (0.01049)	0.27433 (0.01188)	0.19940 (0.05910)	0.37721 **(0.00644)**	0.14815 (0.20602)	*	0.00234 (0.32710)	0.00000 (0.80032)	0.00000 (0.67835)	0.00000 (0.76676)	0.19722 (0.02148)
**Copacabana (Admiralty Bay), King George Island (COP)**	0.30121 **(0.00000)**	0.20420 **(0.00020)**	0.05519 (0.08088)	0.10420 **(0.00119)**	0.28632 **(0.00000)**	0.22873 **(0.00267)**	0.31195 **(0.00000)**	0.20492 (0.01158)	0.00000 (0.41917)	*	0.00000 (0.48758)	0.05467 (0.14523)	0.07275 (0.10237)	0.28589 **(0.00000)**

**Stranger Point, King George Island (SP)**	0.34300<br>**(0.00059)**	0.21259<br>**(0.00673)**	0.00000<br>(0.42679)	0.10256<br>(0.01089)	0.27433<br>(0.01049)	0.19940<br>(0.05871)	0.37721<br>(0.00911)	0.14815<br>(0.21572)	0.00000<br>(0.99990)	0.00000<br>(0.41659)	*	0.00000<br>(0.60539)	0.00000<br>(0.61578)	0.24091<br>(0.02534)
**Bernardo O’Higgins Base (BO)**	0.33193<br>**(0.00010)**	0.20624<br>**(0.00396)**	0.00000<br>(0.36927)	0.09915<br>(0.00980)	0.28556<br>**(0.00228)**	0.20379<br>(0.03227)	0.36842<br>**(0.00307)**	0.15691<br>(0.09583)	0.00000<br>(0.70231)	0.01547<br>(0.25443)	0.00000<br>(0.71211)	*	0.00000<br>(0.67538)	0.11697<br>(0.02614)
**Gabriel González Videla Base (GGV)**	0.47406<br>**(0.00000)**	0.33467<br>**(0.00030)**	0.006308<br>(0.13523)	0.19909<br>**(0.00010)**	0.10105<br>(0.06811)	0.07692<br>(0.45609)	0.19101<br>(0.07613)	0.04328<br>(0.49906)	0.00000<br>(0.61479)	0.08597<br>(0.06376)	0.00000<br>(0.61608)	0.00485<br>(0.38432)	*	0.05175<br>(0.12306)
**Jougla Point (JP)**	0.70966<br>**(0.00000)**	0.58554<br>**(0.00000)**	0.27327<br>**(0.00168)**	0.39345<br>**(0.00000)**	0.00000<br>(0.99990)	0.00000<br>(0.99990)	0.00324<br>(0.50965)	0.00000<br>(0.99990)	0.23112<br>(0.02534)	0.28073<br>**(0.00000)**	0.23112<br>(0.02237)	0.25542<br>**(0.00525)**	0.05560<br>(0.12494)	*

S2B. *TLR5*

Colony	CR	MAR	COU	MO	CB	BR	BI	MT	SIG	COP	SP	BO	GGV	JP
**Crozet Island (CR)**	*	0.10463<br>(0.15701)	0.19875<br>(0.00337)	0.14048<br>(0.00426)	0.42400<br>**(0.00000)**	0.61731<br>**(0.00000)**	0.81884<br>**(0.00000)**	0.65281<br>**(0.00000)**	0.86382<br>**(0.00000)**	0.83274<br>**(0.00000)**	0.93688<br>**(0.00000)**	0.88320<br>**(0.00000)**	0.92114<br>**(0.00000)**	0.96416<br>**(0.00000)**
**Marion Island (MAR)**	0.10463<br>(0.15602)	*	0.27084<br>**(0.00030)**	0.18983<br>**(0.00059)**	0.46395<br>**(0.00000)**	0.66343<br>**(0.00000)**	0.84184<br>**(0.00000)**	0.70960<br>**(0.00000)**	0.90057<br>**(0.00000)**	0.84665<br>**(0.00000)**	0.97033<br>**(0.00000)**	0.91651<br>**(0.00000)**	0.95016<br>**(0.00000)**	0.97642<br>**(0.00000)**
**Courbet Peninsula (COU)**	0.12961<br>(0.02485)	0.27629<br>**(0.00109)**	*	0.00000<br>(0.91209)	0.17852<br>**(0.00050)**	0.33854<br>**(0.00000)**	0.55967<br>**(0.00000)**	0.31502<br>**(0.00000)**	0.50797<br>**(0.00010)**	0.64872<br>**(0.00000)**	0.58133<br>**(0.00000)**	0.54190<br>**(0.00000)**	0.60325<br>**(0.00000)**	0.78796<br>**(0.00000)**
**Pointe du Morne (MO)**	0.10214<br>(0.00713)	0.21189<br>**(0.00000)**	0.00000<br>(0.56351)	*	0.25438<br>**(0.00000)**	0.42963<br>**(0.00000)**	0.56884<br>**(0.00000)**	0.41306<br>**(0.00000)**	0.54117<br>**(0.00000)**	0.63605<br>**(0.00000)**	0.59046<br>**(0.00000)**	0.55817<br>**(0.00000)**	0.59835<br>**(0.00000)**	0.72147<br>**(0.00000)**
**Cow Bay (CB)**	0.28371<br>**(0.00000)**	0.40690<br>**(0.00000)**	0.10025<br>**(0.00000)**	0.09881<br>**(0.00000)**	*	0.05018<br>(0.09811)	0.36548<br>**(0.00000)**	0.05309<br>(0.11880)	0.33306<br>**(0.00000)**	0.45208<br>**(0.00000)**	0.37935<br>**(0.00000)**	0.34106<br>**(0.00000)**	0.39910<br>**(0.00000)**	0.54574<br>**(0.00000)**
**Bull Roads (BR)**	0.39560<br>**(0.00000)**	0.56722<br>**(0.00000)**	0.18264<br>**(0.00000)**	0.17234<br>**(0.00000)**	0.03011<br>(0.09910)	*	0.49814<br>**(0.00000)**	0.00000<br>(0.71864)	0.44207<br>**(0.00188)**	0.58038<br>**(0.00000)**	0.50051<br>**(0.00059)**	0.46193<br>**(0.00119)**	0.53330<br>**(0.00020)**	0.73178<br>**(0.00000)**
**Bird Island (BI)**	0.52955<br>**(0.00000)**	0.66409<br>**(0.00000)**	0.34622<br>**(0.00000)**	0.30010<br>**(0.00000)**	0.13386<br>**(0.00000)**	0.22379<br>**(0.00059)**	*	0.59937<br>**(0.00000)**	0.00978<br>(0.31730)	0.02817<br>(0.12860)	0.05571<br>(0.10425)	0.00778<br>(0.30997)	0.06350<br>(0.10553)	0.17521<br>**(0.00000)**
**Martillo Island (MT)**	0.37560<br>**(0.00000)**	0.57279<br>**(0.00000)**	0.14276<br>**(0.00079)**	0.13905<br>**(0.00010)**	0.04473<br>(0.05455)	0.00000<br>(0.81279)	0.31426<br>**(0.00010)**	*	0.55172<br>**(0.00010)**	0.67565<br>**(0.00000)**	0.62473<br>**(0.00020)**	0.57910<br>**(0.00010)**	0.65295<br>**(0.00000)**	0.83149<br>**(0.00000)**
**Signy Island (SIG)**	0.52861<br>**(0.00000)**	0.71876<br>**(0.00000)**	0.30275<br>**(0.00000)**	0.26750<br>**(0.00000)**	0.10387<br>(0.00762)	0.19030<br>(0.01574)	0.00557<br>(0.31136)	0.27121<br>**(0.00277)**	*	0.08135<br>(0.07187)	0.00000<br>(0.99990)	0.00000<br>(0.67587)	0.00000<br>(0.81546)	0.12682<br>(0.06009)
**Copacabana (Admiralty Bay), King George Island (COP)**	0.42728<br>**(0.00000)**	0.54018<br>**(0.00000)**	0.26264<br>**(0.00000)**	0.23785<br>**(0.00000)**	0.11156<br>**(0.00000)**	0.18623<br>**(0.00109)**	0.06573<br>(0.03732)	0.23511<br>(0.00990)	0.09384<br>(0.05920)	*	0.09581<br>(0.03990)	0.08366<br>(0.04594)	0.11085<br>(0.01465)	0.20084<br>**(0.00000)**
**Stranger Point, King George Island (SP)**	0.66455<br>**(0.00000)**	0.84565<br>**(0.00000)**	0.43966<br>**(0.00000)**	0.36950<br>**(0.00000)**	0.19855<br>**(0.00020)**	0.31769<br>**(0.00267)**	0.01752<br>(0.28591)	0.42613<br>**(0.00040)**	0.00000<br>(0.57499)	0.17399<br>(0.01495)	*	0.00000<br>(0.99990)	0.00000<br>(0.99990)	0.00000<br>(0.99990)
**Bernardo O'Higgins Base (BO)**	0.61772<br>**(0.00000)**	0.79060<br>**(0.00000)**	0.40132<br>**(0.00000)**	0.34057<br>**(0.00000)**	0.16943<br>**(0.00050)**	0.27133<br>**(0.00238)**	0.00117<br>(0.37323)	0.36809<br>**(0.00040)**	0.00000<br>(0.56757)	0.14866<br>(0.01505)	0.00000<br>(0.99990)	*	0.00000<br>(0.79764)	0.05537<br>(0.21948)

**Gabriel González Videla Base (GGV)**	0.58791<br>**(0.00000)**	0.75247<br>**(0.00000)**	0.37912<br>**(0.00000)**	0.32380<br>**(0.00000)**	0.15489<br>**(0.00010)**	0.25607<br>**(0.00248)**	0.00682<br>(0.32373)	0.35394<br>**(0.00020)**	0.00000<br>(0.86714)	0.13331<br>(0.01802)	0.00000<br>(0.81378)	0.00000<br>(0.99990)	*	0.01709<br>(0.18543)
**Jougla Point (JP)**	0.79404<br>**(0.00000)**	0.87888<br>**(0.00000)**	0.65499<br>**(0.00000)**	0.52270<br>**(0.00000)**	0.35294<br>**(0.00000)**	0.53893<br>**(0.00000)**	0.12200<br>**(0.00257)**	0.65751<br>**(0.00000)**	0.14763<br>(0.05821)	0.30787<br>**(0.00000)**	0.00000<br>(0.99990)	0.00582<br>(0.57668)	0.04903<br>(0.11989)	*

S2C. *TLR7*

Colony	CR	MAR	COU	MO	CB	BR	BI	MT	SIG	COP	SP	BO	GGV	JP
**Crozet Island (CR)**	*	0.00000 (0.99990)	0.15385 (0.10049)	0.03575 (0.25166)	0.00000 (0.99990)	0.15385 (0.2206)	0.24153 (0.1199)	0.00000 (0.99990)	0.21932 (0.02099)	0.00575 (0.41451)	0.27835 (0.05821)	0.07738 (0.08643)	0.05127 (0.48193)	0.00000 (0.62479)
**Marion Island (MAR)**	0.00000 (0.99990)	*	0.17066 (0.03406)	0.04320 (0.12395)	0.00000 (0.99990)	0.17065 (0.09524)	0.26718 (0.0988)	0.00000 (0.99990)	0.24170 (0.01584)	0.01208 (0.25423)	0.30218 (0.04445)	0.09237 (0.06316)	0.06381 (0.09049)	0.00013 (0.26186)
**Courbet Peninsula (COU)**	0.13462 (0.09613)	0.15073 (0.03673)	*	0.02638 (0.13038)	0.24508 **(0.00515)**	0.00000 (0.91486)	0.00000 (0.99990)	0.23966 (0.01079)	0.09619 (0.11098)	0.08448 (0.02208)	0.17212 (0.02287)	0.00000 (0.52817)	0.06001 (0.09534)	0.10587 (0.00871)
**Pointe du Morne (MO)**	0.06757 (0.16939)	0.07580 (0.09940)	0.00000 (0.58420)	*	0.07551 (0.02198)	0.00000 (0.59489)	0.00000 (0.60311)	0.06997 (0.03643)	0.13982 (0.02564)	0.02337 (0.11662)	0.18981 (0.00812)	0.00000 (0.50470)	0.02590 (0.20364)	0.03442 (0.04425)
**Cow Bay (CB)**	0.00000 (0.99990)	0.00000 (0.99990)	0.17804 (0.01307)	0.08759 (0.01653)	*	0.21440 (0.01950)	0.30347 (0.03059)	0.00000 (0.99990)	0.31917 **(0.00099)**	0.04066 (0.03465)	0.35766 (0.00941)	0.14082 (0.02594)	0.10513 (0.02277)	0.02675 (0.04277)
**Bull Roads (BR)**	0.15385 (0.21988)	0.17065 (0.08841)	0.00000 (0.99990)	0.00000 (0.86308)	0.18657 (0.0202)	*	0.00000 (0.99990)	0.23965 (0.03346)	0.11188 (0.0694)	0.03171 (0.1389)	0.19988 (0.01792)	0.00000 (0.80804)	0.03355 (0.23849)	0.05073 (0.10286)
**Bird Island (BI)**	0.24153 (0.11771)	0.26718 (0.10019)	0.00000 (0.99990)	0.00000 (0.61113)	0.27337 (0.03158)	0.00000 (0.99990)	*	0.36906 (0.05029)	0.08050 (0.21364)	0.03041 (0.25869)	0.18463 (0.07247)	0.00000 (0.99990)	0.02727 (0.34026)	0.05262 (0.19166)
**Martillo Island (MT)**	0.00000 (0.99990)	0.00000 (0.99990)	0.21679 (0.01238)	0.10671 (0.03554)	0.00000 (0.99990)	0.23965 (0.03772)	0.36906 (0.04960)	*	0.33218 **(0.00307)**	0.03301 (0.08039)	0.39735 (0.01673)	0.15325 (0.02525)	0.11382 (0.03812)	0.01883 (0.13583)
**Signy Island (SIG)**	0.30744 (0.02069)	0.33179 (0.01445)	0.02088 (0.38392)	0.09976 (0.05287)	0.38145 (0.00376)	0.06005 (0.21335)	0.01582 (0.49480)	0.42834 **(0.00267)**	*	0.03625 (0.20493)	0.00000 (0.99990)	0.00039 (0.44204)	0.00000 (0.68350)	0.05056 (0.16315)
**Copacabana (Admiralty Bay), King George Island (COP)**	0.05824 (0.12672)	0.06574 (0.10247)	0.00000 (0.51431)	0.00125 (0.34294)	0.07694 (0.00802)	0.00000 (0.47381)	0.00000 (0.51579)	0.09310 (0.02079)	0.03264 (0.16563)	*	0.05349 (0.14266)	0.00000 (0.83318)	0.00000 (0.86249)	0.00000 (0.57965)
**Stranger Point, King George Island (SP)**	0.27835 (0.05693)	0.30218 (0.04792)	0.03784 (0.22651)	0.09551 (0.04534)	0.33172 (0.00931)	0.08347 (0.14523)	0.05636 (0.26324)	0.39735 (0.01832)	0.00000 (0.99990)	0.01031 (0.37036)	*	0.06359 (0.19364)	0.00000 (0.60915)	0.05583 (0.14642)
**Bernardo O'Higgins Base (BO)**	0.14089 (0.08128)	0.15866 (0.07019)	0.00000 (0.99990)	0.00000 (0.76052)	0.17845 (0.02544)	0.00000 (0.99990)	0.00000 (0.99990)	0.23146 (0.02584)	0.00000 (0.57440)	0.00000 (0.82398)	0.00000 (0.43817)	*	0.00000 (0.83586)	0.00000 (0.48965)

**Gabriel González Videla Base (GGV)**	0.10256 (0.21968)	0.11742 (0.08841)	0.00000 (0.68023)	0.00000 (0.45857)	0.13061 (0.03425)	0.00000 (0.7821)	0.00000 (0.58202)	0.17796 (0.03861)	0.00000 (0.58103)	0.00000 (0.99990)	0.00000 (0.61053)	0.00000 (0.99990)	*	0.00000 (0.85031)
**Jougla Point (JP)**	0.05241 (0.11712)	0.05952 (0.09356)	0.00000 (0.39155)	0.00830 (0.22443)	0.06981 (0.01099)	0.00696 (0.32888)	0.00000 (0.38491)	0.08498 (0.01634)	0.03980 (0.14929)	0.00000 (0.76933)	0.01196 (0.36442)	0.00000 (0.70765)	0.00000 (0.93100)	*

S2D. HVR1

Colony	CR	MAR	COU	MO	CB	BR	BI	MT	SIG	COP	SP	JP
**Crozet Island (CR)**	*	0.00000<br>(0.56638)	0.98071<br>(0.03178)	0.97191<br>**(0.00000)**	0.96572<br>**(0.00000)**	0.97018<br>**(0.00040)**	0.97094<br>**(0.00000)**	0.96879<br>**(0.00149)**	0.96497<br>**(0.00149)**	0.9557<br>**(0.00000)**	0.94835<br>**(0.00010)**	0.96015<br>**(0.00000)**
**Marion Island (MAR)**	0.00000<br>(0.66974)	*	0.98950<br>(0.02376)	0.97523<br>**(0.00000)**	0.97019<br>**(0.00000)**	0.97819<br>**(0.00020)**	0.97591<br>**(0.00000)**	0.97818<br>**(0.00079)**	0.97557<br>**(0.00069)**	0.96038<br>**(0.00000)**	0.95916<br>**(0.00040)**	0.96415<br>**(0.00000)**
**Courbet Peninsula (COU)**	0.35052<br>(0.11405)	0.39100<br>(0.02455)	*	0.29453<br>(0.01287)	0.94336<br>(0.00683)	0.96564<br>(0.02673)	0.96057<br>**(0.00594)**	0.96480<br>(0.04821)	0.95757<br>(0.04831)	0.92969<br>**(0.00475)**	0.90586<br>(0.03534)	0.93788<br>**(0.00297)**
**Pointe du Morne (MO)**	0.08579<br>**(0.00238)**	0.11761<br>**(0.00030)**	0.21195<br>(0.02079)	*	0.92836<br>**(0.00000)**	0.92743<br>**(0.00000)**	0.92721<br>**(0.00000)**	0.92737<br>**(0.00000)**	0.91436<br>**(0.00010)**	0.91865<br>**(0.00000)**	0.90576<br>**(0.00000)**	0.92253<br>**(0.00000)**
**Cow Bay (CB)**	0.11875<br>**(0.00257)**	0.15084<br>**(0.00099)**	0.28772<br>(0.02138)	0.07047<br>**(0.00010)**	*	0.10968<br>(0.03356)	0.7874<br>**(0.00000)**	0.28031<br>**(0.00208)**	0.74375<br>**(0.00000)**	0.71836<br>**(0.00000)**	0.70848<br>**(0.00000)**	0.73538<br>**(0.00000)**
**Bull Roads (BR)**	0.14286<br>(0.01188)	0.17963<br>**(0.00564)**	0.35052<br>(0.10781)	0.08579<br>**(0.00248)**	0.08636<br>(0.03257)	*	0.84043<br>**(0.00000)**	0.28029<br>(0.03346)	0.82431<br>**(0.00158)**	0.75894<br>**(0.00000)**	0.74524<br>**(0.00069)**	0.77946<br>**(0.00000)**
**Bird Island (BI)**	0.12855<br>(0.00653)	0.16014<br>**(0.00059)**	0.29812<br>(0.01228)	0.07930<br>**(0.00000)**	0.10888<br>**(0.00010)**	0.12855<br>(0.00624)	*	0.84374<br>**(0.00000)**	0.32908<br>**(0.00030)**	0.24287<br>**(0.00000)**	0.25257<br>**(0.00020)**	0.22048<br>**(0.00000)**
**Martillo Island (MT)**	0.25878<br>**(0.00465)**	0.29433<br>**(0.00218)**	0.55224<br>(0.10375)	0.17732<br>**(0.00040)**	0.21433<br>**(0.00238)**	0.10862<br>(0.12751)	0.22363<br>**(0.00168)**	*	0.80733<br>(0.00762)	0.74466<br>**(0.00000)**	0.70428<br>**(0.00287)**	0.76889<br>**(0.00000)**
**Signy Island (SIG)**	0.07667<br>(0.10969)	0.11833<br>(0.05465)	0.28571<br>(0.04663)	0.02460<br>(0.19840)	0.05851<br>(0.11118)	0.07667<br>(0.10920)	0.06939<br>(0.07970)	0.20000<br>(0.07801)	*	0.01749<br>(0.29324)	0.03152<br>(0.26304)	0.01385<br>(0.29621)
**Copacabana (Admiralty Bay), King George Island (COP)**	0.08074<br>**(0.00208)**	0.11247<br>**(0.00010)**	0.23804<br>(0.01861)	0.03770<br>**(0.00000)**	0.06588<br>**(0.00020)**	0.08074<br>**(0.00257)**	0.07469<br>**(0.00000)**	0.17159<br>**(0.00030)**	0.01948<br>(0.22285)	*	0.00000<br>(0.53480)	0.01858<br>(0.15929)
**Stranger Point, King George Island (SP)**	0.07363<br>(0.10019)	0.11346<br>(0.04574)	0.26829<br>(0.03198)	0.02384<br>(0.16553)	0.05657<br>(0.07574)	0.07363<br>(0.10217)	0.06709<br>(0.05653)	0.18978<br>**(0.00119)**	0.00000<br>(0.99990)	0.01097<br>(0.31829)	*	0.06616<br>(0.08742)
**Jougla Point (JP)**	0.09405<br>**(0.00475)**	0.12503<br>**(0.00069)**	0.25198<br>(0.01376)	0.05014<br>**(0.00030)**	0.07823<br>**(0.00010)**	0.09405<br>**(0.00465)**	0.06869<br>**(0.00050)**	0.18393<br>**(0.00149)**	0.01765<br>(0.26225)	0.02247<br>(0.04208)	0.03323<br>(0.14088)	*

Supplementary Table S3. Census population size (number of breeding pairs) applied to analyses related to each colony, based on best available census data for each island group or region, with census year and reference information.

Colony	Census Region	Census Population (breeding pairs)	Year	Reference / Notes
CR	Crozet Archipelago	9,000	1994	Jouventin, 1994.
MAR	Marion Island	600	2012/13	Crawford, et al. 2014.
COU	Kerguelen Islands	30,000	1988	Weimerskirch, et al. 1988. (*Lescroel, and Bost, 2006. show significant decrease in 2002 of Kerguelen population, but was localized to the sampled portion of the archipelago, leading us to use 30,000 as the lower range number for Weimerskirch, et al. 1998's estimate of 30-40,000)
MO				
CB	Falkland/ Malvinas Islands	132,321	2010	Baylis, et al. 2013.
BR				
BI	South Georgia	98,867	1996	Trathan, et al. 1996.
MT	Martillo Island	12	2006/07	Ghys, et al. 2008.
SIG	South Orkney Islands	10,760	2013	Lynch 2013.
COP	South Shetland Islands and Western Antarctic Peninsula	94,751	2013	Lynch 2013.
SP				
BO				
GGV				
JP				

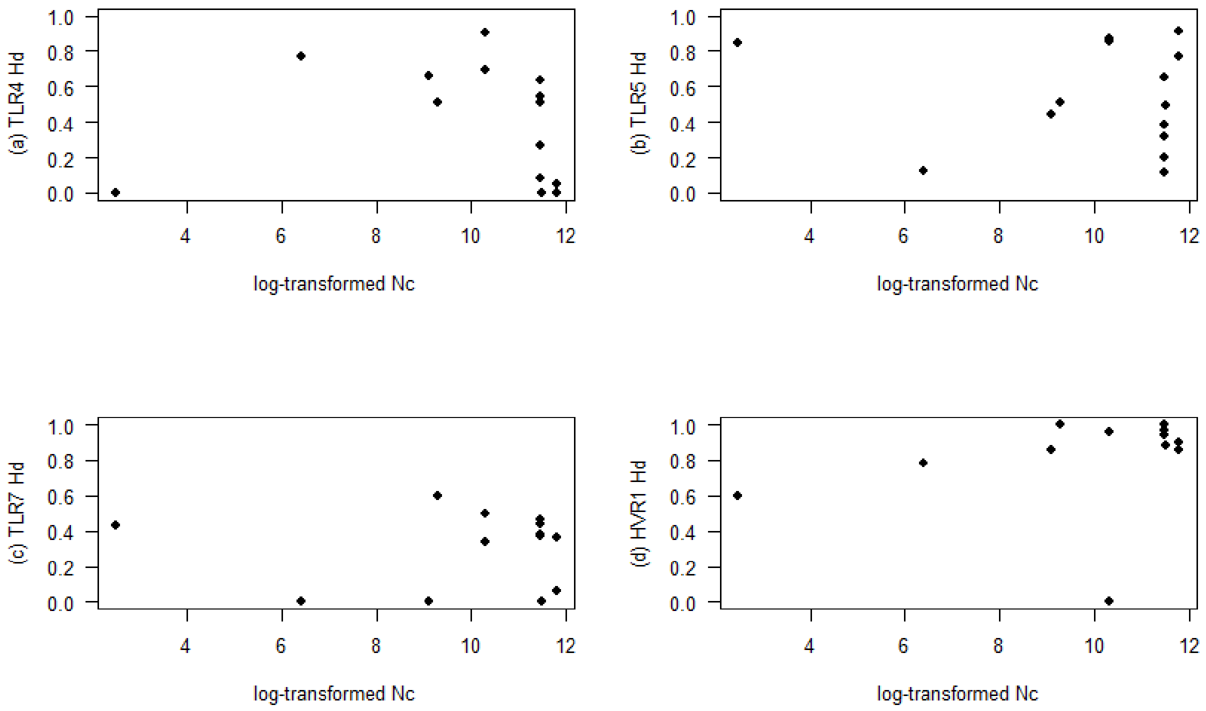

Supplementary Figure S1. Haplotype diversity (H_d) for (a) *TLR4*, (b), *TLR5*, (c) *TLR7*, and (d) mtDNA HVR1 in relation to census population size across Gentoo penguin colonies. Census population sizes and their source publications used for analyses are found in Supplementary Table S3.

Supplementary Table S4. Spearman's rank correlations and *p* values calculated for each haplotype diversity Hd vs. census population size comparison depicted in Supplementary Figure S1.

Locus	Spearman's rank correlation	*p*
TLR4	-0.5029245	0.06679
TLR5	0.09462364	0.7476
TLR7	-0.1380377	0.6379
HVR1	N/A	0.3263

Supplementary Table S5A. Mantel Test results for Isolation by Distance for each locus, using F_{ST} (Weir and Cockerham 1984) values against a matrix of geographic distance (km), with significance evaluated using 10,000 permutations in Arlequin.

**Mantel Test Locus**	**Correlation Coefficient (R)**	***P* value**
TLR4	0.514998	0.0011
TLR5	0.592887	0.0010
TLR7	0.026662	0.3677
**HVR1**	0.311577	0.0153

Supplementary Table S5B. Pairwise geographic distances (most direct distance by sea during summer sea ice extent, in km) used for Mantel's test, derived from Google Earth v7.3.2.5776.

**Colony**	**CR**	**MAR**	**COU**	**MO**	**CB**	**BR**	**BI**	**MT**	**SIG**	**COP**	**SP**	**BO**	**GGV**	**JP**
**Crozet Island (CR)**	0													
**Marion Island (MAR)**	960	0												
**Courbet Peninsula (COU)**	1577	2461	0											
**Pointe du Morne (MO)**	1565	2449	14	0										
**Cow Bay (CB)**	7190	6481	7850	7837	0									
**Bull Roads (BR)**	7185	6504	7833	7819	168	0								
**Bird Island (BI)**	5958	5204	6786	6773	1359	1432	0							
**Martillo Island (MT)**	7354	6743	7915	7903	792	639	1892	0						
**Signy Island (SIG)**	5955	5315	6565	6555	1282	1258	901	1437	0					
**Copacabana (Admiralty Bay), King George Island (COP)**	6419	5848	6978	6970	1275	1173	1501	1010	705	0				
**Stranger Point, King George Island (SP)**	6421	5849	6979	6971	1262	1161	1506	995	711	13	0			
**Bernardo O'Higgins Base (BO)**	6402	5804	6936	6928	1351	1248	1536	1085	708	130	125	0		
**Gabriel González Videla Base (GGV)**	6705	6107	7237	7229	1522	1414	1837	1147	1009	372	360	302	0	
**Jougla Point (JP)**	6719	6138	7264	7256	1543	1435	1861	1170	1035	400	391	328	45	0

Supplementary Table S6A. Log-likelihood comparisons for PAML (Yang 1997; Yang 2007) analysis of *TLR4*.

**Model**	**lnL**	**2(lnL$_1$-lnL$_0$)**	**df**	***p-value***
**M1a**	-3605.474038	8.33452799999986	2	0.015494595
**M2a**	-3597.13951			
**M7**	-3605.717024	8.33604300000025	2	0.015482863
**M8**	-3597.380981			
**M8a**	-3605.474029	8.09304800000018	2	0.01748304
**M8**	-3597.380981			

Supplementary Table S6B. Bayes Empirical Bayes (Yang, et al. 2005) analysis of *P. papua TLR4* sites under positive selection with posterior probability >0.90. Sites obtained from *codeml* M2a.

**Amino acid pos.**	**Amino acid**	**Pr(ω>1)**	**Mean ω ± SE**
12	V	0.932	7.669 ± 2.761
236	I	0.932	7.669 ± 2.761
659	A	0.933	7.671 ± 2.759

Supplementary Table S6C. Bayes Empirical Bayes analysis of *P. papua TLR4* sites under positive selection with posterior probability >0.90. Sites obtained from *codeml* M8.

**Amino acid pos.**	**Amino acid**	**Pr(ω>1)**	**Mean ω ± SE**
12	V	0.947	1.461 ± 0.163
82	N	0.936	1.485 ± 0.000
236	I	0.947	1.461 ± 0.163
316	C	0.936	1.485 ± 0.000
445	Q	0.936	1.485 ± 0.000
659	A	0.947	1.461 ± 0.163

Supplementary Table S6D. Log-likelihood comparisons for PAML analysis of *TLR5*.

**Model**	**lnL**	**2(lnL$_1$-lnL$_0$)**	**df**	***p-value***
**M1a**	-3929.201728	102.078912	2	6.821e-23
**M2a**	-3878.162272			
**M7**	-3929.323036	102.311604	2	6.072e-23
**M8**	-3878.167234			
**M8a**	-3928.465238	100.596008	2	7.16e-23
**M8**	-3878.167234			

Supplementary Table S6E. Bayes Empirical Bayes analysis of *P. papua TLR5* sites under positive selection with posterior probability >0.90. Sites obtained from *codeml* M2a.

**Amino acid pos.**	**Amino acid**	**Pr(ω>1)**	**Mean ω ± SE**
10	Y	1.000	9.645 ± 0.504
285	M	0.980	9.472 ± 1.320
442	A	0.998	9.631 ± 0.609
535	S	0.981	9.482 ± 1.286
667	S	1.000	9.643 ± 0.514
698	M	0.980	9.469 ± 1.329
747	L	0.985	9.518 ± 1.163
788	C	0.982	9.492 ± 1.255
845	V	1.000	9.645 ± 0.503

Supplementary Table S6F. Bayes Empirical Bayes analysis of *P. papua TLR5* sites under positive selection with posterior probability >0.90. Sites obtained from *codeml* M8a.

**Amino acid pos.**	**Amino acid**	**Pr(ω>1)**	**Mean ω ± SE**
10	Y	1.000	8.947 ± 1.200
285	M	0.989	8.855 ± 1.487
442	A	0.999	8.941 ± 1.219
535	S	0.989	8.860 ± 1.472
667	S	1.000	8.946 ± 1.202
698	M	0.988	8.853 ± 1.490
747	L	0.991	8.878 ± 1.421
788	C	0.990	8.865 ± 1.459
845	V	1.000	8.947 ± 1.200

Supplementary Table S7. Physiochemical distances between amino acid variants at positively selected sites in TLR4 and TLR5, as given by several authors, detailed in the lower table. Red colors indicate a greater physiochemical distance, while green colors indicate a lower physiochemical distance. *Epstein's distance has an element of directionality, so both directions are given: Epstein_1 = variant_1 → variant_2; Epstein_2 = variant_2 → variant_1.

TLR	nt_site	aa_site	variant_1	variant_2	Grantham [1]	Sneath [2]	Epstein_1 [3]	Epstein_2 [3]	Miyata [4]	Urbina [5]
TLR4	35	12	V	A	64	12	0.4	0.41	1.85	3.125
TLR4	706	236	V	I	29	7	0.03	0.05	0.85	0.873
TLR4	1977	659	A	T	58	20	0.41	0.4	0.9	1.795
TLR5	29	10	C	Y	194	34	0.25	0.13	2.38	3.591
TLR5	854	285	T	M	81	25	0.8	0.8	1.86	2.404
TLR5	1325	442	A	V	64	12	0.41	0.4	1.85	3.125
TLR5	1604	535	S	T	58	12	0.03	0.03	0.89	1.697
TLR5	2000	667	F	S	155	25	0.81	0.81	3.45	4.544
TLR5	2094	698	I	M	10	22	0.05	0.03	0.29	1.565
TLR5	2239	747	V	L	32	9	0.05	0.03	0.91	0.831
TLR5	2362	788	S	C	112	13	0.6	0.6	2.84	2.758
TLR5	2533	845	V	I	29	7	0.05	0.03	0.85	0.873

Number	Criteria	Range	Reference
[1]	composition, polarity, molecular volume	5(low)-215(high)	Grantham (1974)
[2]	134 categories of activity and structure	5(low)-45(high)	Sneath (1966)
[3]*	polarity, size	0(low)-1.02(high)	Epstein (1967)
[4]	volume, polarity	0.06(low)-5.13(high)	Miyata (1979)
[5]	volume, bulkiness, polarity, isoelectric point, hydrophobicity, surface area accessible to water, fraction of accessible area lost when protein folds	1.031(low)-7.08(high)	Urbina, et al. (2006)

Supplementary Table S8. Positions of Gentoo penguin positively selected sites with respect to sites of known function in other vertebrates. The position and name of the corresponding chicken amino acid is given for each Gentoo TLR position, as chicken TLRs are typically the best-studied and best-annotated TLRs. "other_literature" denotes a study in which the described sites have also been found to be positively selected in a particular taxon (references are given below). "function" denotes the potential for change in function, due to proximity to sites of known function. "N/A" indicates that the Gentoo TLR5 open reading frame begins upstream of other known TLRs, so no functional information is known.

TLR	nt_site	aa_site	variant_1	variant_2	chicken_posn	chicken_aa	other_literature	function
TLR4	35	12	V	A	6	A	Velová, et al. 2018	No
TLR4	706	236	V	I	230	I	No	No
TLR4	1977	659	A	T	653	A	No	No
TLR5	29	10	C	Y	N/A	N/A	N/A	N/A
TLR5	854	285	T	M	267	M	No	Adjacent to two residues in flagellin-binding interface B (266,268) Yoon, et al. 2012; Song, et al. 2017
TLR5	1325	442	A	V	424	A	Velová, et al. 2018; Grueber, et al. 2014	No
TLR5	1604	535	S	T	517	S	No	No
TLR5	2000	667	F	S	649	S	No	No
TLR5	2094	698	I	M	680	L	Velová, et al. 2018	No
TLR5	2239	747	V	L	729	L	No	No
TLR5	2362	788	S	C	770	C	No	No
TLR5	2533	845	V	I	827	I	No	No

TLR4

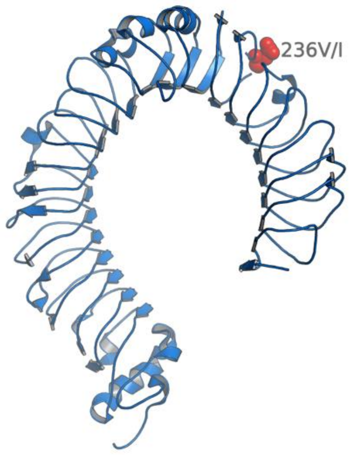

TLR5

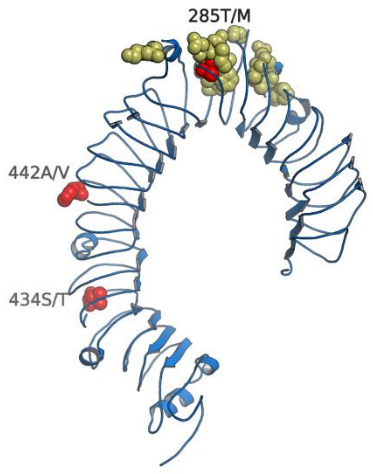

Supplementary Figure S2. Positions of positively selected sites in Gentoo penguin TLR4 and TLR5 ectodomains. Positively selected sites from PAML analysis (highlighted in red) are superimposed on homologous sites from the crystal structure of mouse TLR4 ectodomain (PDB: 2Z64) and a homology model of human TLR5 ectodomain (PDB: 3J0A). Further positively selected sites in the signal peptide of TLR4 (12V/A) and TLR5 (10C/Y), as well as TMD/TIR domain sites (TLR4: 659A/T; TLR5: 667F/S, 698I/M, 747V/L, 788S/C, 845V/I) were omitted because the residues are not included in the available model structure, or were of low confidence. Relative positions of these residues are depicted schematically in main text Figure 6. Site 285T/M is located within interface-B of the flagellin binding site (Yoon, et al. 2012; Song, et al. 2017), which is highlighted in yellow. No other positively selected sites were found to be proximal to sites of known function in other vertebrates.

Supplementary Table S9. *In silico* prediction of change of function of positively selected sites using the homology-based methods SIFT (Ng and Henikoff 2003) and PolyPhen-2 (Adzhubei, et al. 2013). Both SIFT and PolyPhen-2 are directional, so the scores for each variant 1 → variant 2 and variant 2 → variant 1 are given. Red colours indicate the change is likely to be of high consequence, while green colours indicate milder consequences.

TLR	nt_site	aa_site	variant_1	variant_2	SIFT				Polyphen2					
					v1→v2		v2→v1		v1→v2			v2→v1		
					score	MSC	score	MSC	score	sensitivity	specificity	score	sensitivity	specificity
TLR4	35	12	V	A	0.15	3.34	0.58	3.38	N/A	N/A	N/A	0.997	0.410	0.980
TLR4	706	236	V	I	1.00	3.20	0.18	3.21	0.001	0.990	0.150	0.049	0.940	0.830
TLR4	1977	659	A	T	0.20	3.04	0.25	3.05	0.810	0.840	0.930	0.000	1.000	0.000
TLR5	29	10	C	Y	0.00	4.32	0.00	4.32	0.004	0.970	0.590	N/A	N/A	N/A
TLR5	854	285	T	M	0.43	3.36	0.10	3.36	0.000	1.000	0.000	0.990	0.720	0.970
TLR5	1325	442	A	V	0.51	3.55	0.72	3.36	0.165	0.920	0.870	0.000	1.000	0.000
TLR5	1604	535	S	T	0.38	3.36	0.82	3.36	0.253	0.910	0.880	0.000	1.000	0.000
TLR5	2000	667	F	S	0.34	3.36	0.01	3.36	0.000	1.000	0.000	0.998	0.270	0.990
TLR5	2094	698	I	M	0.06	3.36	0.45	3.36	0.009	0.960	0.770	0.005	0.970	0.740
TLR5	2239	747	V	L	1.00	3.36	0.00	3.36	0.000	1.000	0.000	0.000	1.000	0.000
TLR5	2362	788	S	C	1.00	3.36	0.00	3.36	0.000	1.000	0.000	0.992	0.700	0.970
TLR5	2533	845	V	I	0.45	3.36	1.00	3.36	0.024	0.950	0.810	0.000	1.000	0.000

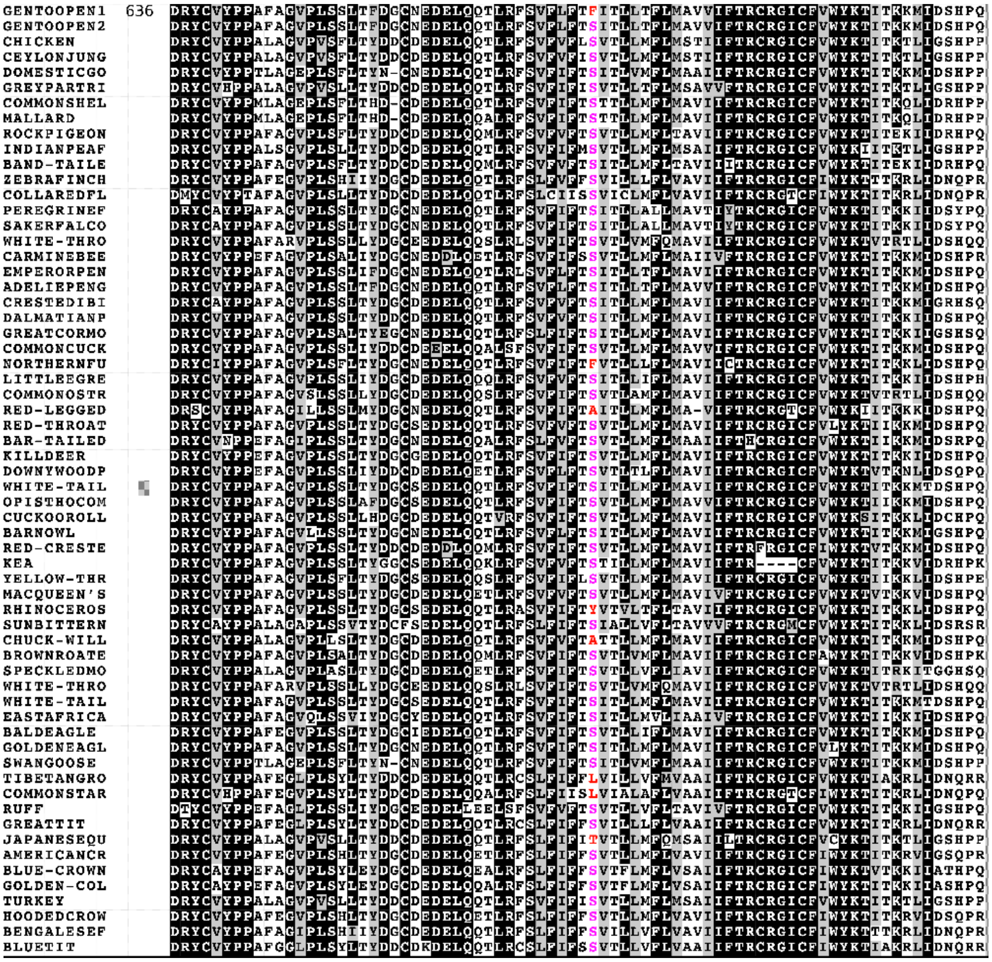

Supplementary Figure S3. Alignment of the transmembrane region and surrounding sequence of available avian TLR5 amino acid sequences. The conserved serine residue is highlighted in pink and any variant amino acids are highlighted in red.

Supplementary Table S10. Diversity statistics of Gentoo penguin (*Pygoscelis papua*) vs. avian Toll-like receptors from published studies. Chicken diversity data was drawn directly from Świderská, et al. (2018), while grey partridge study sequences were obtained from GenBank along with supplementary frequency data for analysis in DnaSP 6.12.10 as in main text methods to obtain comparable diversity measures.

Gene	Species	Length (bp)	*k*	H_d ± SD	π ± SD	UPV	A
TLR4	Gentoo penguin	2550	1.550	0.478 ± 0.035	0.00061 ± 0.00005	9	21
	Domestic chicken	2532	6.540	0.947 ± 0.010	0.00258 ± 0.00005	24	70
	Grey partridge	2529	3.063	0.863 ± 0.063	0.00121 ± 0.00018	5	10
TLR5	Gentoo penguin	2643	3.919	0.781 ± 0.022	0.00148 ± 0.00006	32	46
	Domestic chicken	2586	2.866	0.854 ± 0.013	0.00111 ± 0.00004	10	22
	Grey partridge	2589-2592	1.053	0.747 ± 0.076	0.00041 ± 0.00008	4	6
TLR7	Gentoo penguin	3126	0.327	0.296 ± 0.034	0.00010 ± 0.00001	8	10
	Domestic chicken	3180	3.363	0.849 ± 0.017	0.00106 ± 0.00005	13	26
	Grey partridge	3141	1.753	0.532 ± 0.100	0.00056 ± 0.00013	3	3

k = average number of nucleotide differences between alleles; Hd = haplotype diversity; SD: standard deviation; π: nucleotide diversity (average number of nucleotide differences per site between two sequences); UPV: number of unique protein variants; A: number of alleles.

Notes: To compare diversity levels in our study population with those of other species, we sought datasets that sequenced *TLR4*, *TLR5*, and *TLR7* in birds. Because diversity measures can be biased by gene length and whether sequenced fragments are under selection, we restricted our comparison only to studies that sequenced the full length of these genes: a study in 110 domestic chickens (*Gallus gallus domesticus*, Świderská, et al. 2018) and a study of 10 European grey partridges (*Perdix perdix*, Vinkler, et al. 2015). The difference in gene length among these species is minimal enough to allow comparison (*TLR4*: <0.01%, *TLR5*: 2.15%, *TLR7*: 1.69%). However, sampling strategy differs significantly between studies, and also affects diversity measures (i.e. chicken study sampled few individuals from 25 distinct farmed breeds, partridge study sampled a small geographic subset of the species, while Gentoo penguin study had varying numbers of samples from multiple wild populations). Given the differences in sampling strategy, only relative statements comparing TLRs within a species/study are appropriate, and care should be given to drawing conclusions from interspecific comparisons.

Supplementary Table S11. Sampling sites, location details, and number of samples obtained from Gentoo penguins for immunogenetic analysis.

**Sampling Site Name**	**Region**	**Latitude**	**Longitude**	**Sample size**			
				mtDNA	*TLR4*	*TLR5*	*TLR7*
**Crozet Island (CR)**	Crozet Is.	46.4298 S	50.4046 E	7	7	7	7
**Marion Island (MAR)**	Prince Edward Is.	46.8872 S	37.8671 E	8	8	8	8
**Courbet Peninsula (COU)**	Kerguelen Is.	49.2811 S	70.5410 E	2	7	7	7
**Pointe du Morne (MO)**	Kerguelen Is.	49.3805 S	70.4423 E	20	15	20	18
**Cow Bay (CB)**	Falkland/Malvinas Is.	51.4343 S	57.8506 W	16	19	18	17
**Bull Roads (BR)**	Falkland/Malvinas Is.	52.3380 S	59.3633 W	7	7	7	7
**Bird Island (BI)**	South Georgia	54.0094 S	38.0631 W	17	18	12	13
**Martillo Island (MT)**	Tierra del Fuego	54.9083 S	67.3833 W	5	5	5	4
**Signy Island (SIG)**	South Orkney Is.	60.6747 S	45.6278 W	5	5	5	5
**Copacabana (Admiralty Bay), King George Island (COP)**	South Shetland Is.	62.1792 S	58.4472 W	21	22	21	21
**Stranger Point, King George Island (SP)**	South Shetland Is.	62.2375 S	58.5939 W	6	5	5	5
**Bernardo O'Higgins Base (BO)**	Western Antarctic Peninsula	63.3207 S	57.8987 W	0	6	6	6
**Gabriel González Videla Base (GGV)**	Western Antarctic Peninsula	64.8238 S	62.8581 W	0	7	7	7
**Jougla Point (JP)**	Western Antarctic Peninsula	64.8270 S	63.4932 W	24	24	24	23
			**Total**	138	155	152	148

Supplementary Table S12. List of primers used for PCR amplification and sequencing of Gentoo penguin TLR genes, along with reaction conditions.

Gene	Description[1] and (Primer Names)	Primer Sequences	Ta (°C)	Product Size	Polymerase / reaction volume / PCR program
TLR4	Exon 1 (PengTLR4_Ex1_F2 + PengTLR4_Ex1_R2_long)	F: CTCACTCCAGGTCCTTGAGTG<br>R: ACTGCAGTACCCTCAGAGGT	55	419 bp	Qiagen *Taq* PCR Master Mix Kit (12 µL)<br>94° C / 3:00<br>40 cycles:<br>94° C / 0:45<br>55° C / 0:45<br>72° C / 1:00<br>72° C / 10:00
TLR4	Exon 2 (PengTLR4_Ex2_F2 + (PengTLR4_Ex2_R1)	F: TGCTGCTTTCCTGACAGTGT<br>R: AGCCAAGACCAACAGACACC	55	358 bp	Qiagen *Taq* PCR Master Mix Kit (12 µL)<br>94° C / 3:00<br>40 cycles:<br>94° C / 0:45<br>55° C / 0:45<br>72° C / 1:00<br>72° C / 10:00
TLR4	Exon 3 (Start) (PengTLR4_Ex3_Start_F + PengTLR4_Ex3_Start_R2)	F: AAGGGACAGTGTTGCATGCA<br>R: CACTCAAATCTACAACCTCCAGG	55	945 bp	Qiagen *Taq* PCR Master Mix Kit (12 µL)<br>94° C / 3:00<br>40 cycles:<br>94° C / 0:45<br>55° C / 1:00<br>72° C / 1:00<br>72° C / 10:00
TLR4	Exon 3 (Middle) (PengTLR4_Ex3_Mid_F1 + PengTLR4_Ex3_MidR2)	F: GGCAACGTCTCTAGTATTCGGT<br>R: TGCAGGTGTGTGGCATATCA	55	932 bp	Qiagen *Taq* PCR Master Mix Kit (12 µL)<br>94° C / 3:00<br>40 cycles:<br>94° C / 0:45<br>55° C / 1:00<br>72° C / 1:00<br>72° C / 10:00
TLR4	Exon 3 (End)[2] (PengTLR4_Ex3_Mid_F1 + PengTLR4_Ex3_EndR2 or PengTLR4_Ex3_EndR)	F: GGCAACGTCTCTAGTATTCGGT<br>R: TGCCTTCTAGCAGGACTCCT<br>or<br>R: ACTTCTGAGCACCAGCCATC	50	1563 bp or 1671 bp	Qiagen *Taq* PCR Master Mix Kit (12 µL)<br>94° C / 3:00<br>40 cycles:

					94° C / 0:45<br>50° C / 1:00<br>72° C / 2:00<br>72° C / 10:00
TLR5	TLR5 (A)<br>(TLR5_full_new_F +<br>TLR5_1000_R)	F:<br>AGTGTGACATTAGTCTTTTGTTCTACA<br><br>R: AGCCAAATCCCGAACCCATT	64	1021 bp	NEB Phusion Hot Start Flex DNA Polymerase (25 µL)<br>98° C / 3:00<br>40 cycles:<br>98° C / 0:30<br>64° C / 0:30<br>72° C / 1:00<br>72° C / 10:00
TLR5	TLR5 (B)<br>(Gentoo_TLR5_sequencing_2_F<br>+ Gentoo_TLR5_full_R)	F: ACCTTAGTGACAACGGCTGG<br><br>R: GTGTTCATTCCTGCCATGGC	68	1948 bp	NEB Phusion Hot Start Flex DNA Polymerase (25 µL)<br>98° C / 3:00<br>40 cycles:<br>98° C / 0:30<br>68° C / 0:30<br>72° C / 2:00<br>72° C / 10:00
TLR5	TLR5 (C)<br>(Gentoo_TLR5_sequencing_3_F<br>+ Gentoo_TLR5_full_R)	F: TCCCCTGAGCCTGAAGTCTT<br><br>R: GTGTTCATTCCTGCCATGGC	69	962 bp	NEB Phusion Hot Start Flex DNA Polymerase (25 µL)<br>98° C / 3:00<br>40 cycles:<br>98° C / 0:30<br>69° C / 0:30<br>72° C / 1:00<br>72° C / 10:00
TLR7	TLR7 Coding Exon (A)<br>(TLR7_StartF_212 +<br>TLR7_AR_882)	F: AGCCAGTGTGAGAGACAGGA<br><br>R: ACGGTAGTAACAGTTCTGTC	62	670 bp	NEB Phusion Hot Start Flex DNA Polymerase (25 µL)<br>98° C / 3:00<br>40 cycles:<br>98° C / 0:30<br>62° C / 0:30<br>72° C / 0:30<br>72° C / 10:00
TLR7	TLR7 Coding Exon (B)<br>(TLR7_BF_832 +<br>TLR7_BR_1733)	F: GAGCTAGGAAACATAGAAAT<br><br>R: TCATCATACCTGAAATAATG	55	901 bp	NEB Phusion Hot Start Flex DNA Polymerase (25 µL)<br>98° C / 3:00<br>40 cycles:<br>98° C / 0:30

					55° C / 0:30<br>72° C / 0:45<br>72° C / 10:00
***TLR7*[3]**	TLR7 Coding Exon (C)<br>(TLR7_midF_1512 +<br>TLR7_midR_2741)	F: AAATCTAACAGTGTTGGATC<br>R: GTCACTTGAGTCTGATTGAT	50	1229 bp	NEB Phusion Hot Start Flex DNA Polymerase (25 µL)<br>98° C / 3:00<br>40 cycles:<br>98° C / 0:30<br>50° C / 0:30<br>72° C / 0:30<br>72° C / 10:00
TLR7	TLR7 Coding Exon (D)<br>(TLR7_DF_2613 +<br>TLR7_End_DR_3597)	F: AATTAAGAAATCTAGCTTCC<br>R: TTTAGGGTACAATATCACAA	54	984 bp	NEB Phusion Hot Start Flex DNA Polymerase (25 µL)<br>98° C / 3:00<br>40 cycles:<br>98° C / 0:30<br>54° C / 0:45<br>72° C / 0:30<br>72° C / 10:00

[1] Longer exons are broken up into fragments due to the practical limitations of Sanger sequencing. Fragments are listed sequentially in the table, with sufficient overlaps to allow for sequence assembly. The descriptors Start/Middle/End or A/B/C/D also reflect this ordering of fragments of the gene.

[2] Certain samples yielded cleaner PCR amplicons and sequencing results with either of these two Reverse primers, so both are listed. PCRs were conducted under the same conditions for both combinations.

[3] This fragment was GC-rich and required Phusion GC buffer (NEB) rather than HF buffer for successful PCR amplification. The presence of a secondary structure here required repeat bi-directional sequencing for most samples.

Supplementary Information References

Adzhubei I, Jordan DM, Sunyaev SR. 2013. Predicting functional effect of human missense mutations using PolyPhen-2. Current Protocols in Human Genetics 76(1)7.20.1-7.20.41.

Baylis AMM, Crofts S, Wolfaardt AC. 2013. Population trends of Gentoo Penguins Pygoscelis papua breeding at the Falkland Islands. Marine Ornithology 41: 1–5.

Carvajal-Rodriguez A, de Uña-Alvarez J. 2011. Assessing Significance in High-Throughput Experiments by Sequential Goodness of Fit and q-Value Estimation. PLoS One 6:e24700.

Crawford RJM, Dyer BM, Upfold L, Makhado AB. 2014. Congruent, decreasing trends of Gentoo Penguins and Crozet Shags at sub-Antarctic Marion Island suggest food limitation through common environmental forcing. African Journal of Marine Science 36: 225–231.

Epstein CJ. 1967. Non-randomness of ammo-acid changes in the evolution of homologous proteins. Nature 215(5099):355-359.

Ghys MI, Raya Rey A, Schiavini A. 2008. Population trend and breeding biology of Gentoo penguin in Martillo Island, Tierra Del Fuego, Argentina. Waterbirds 31(4):625-631.

Grantham R. 1974. Amino acid difference formula to help explain protein evolution, Science. 185(4154):862-864

Grueber CE, Wallis GP, Jamieson IG. 2014. Episodic Positive Selection in the Evolution of Avian Toll-Like Receptor Innate Immunity Genes. Plos One 9.

Jouventin, P. 1994. Les populations d'oiseaux marins des T.A.A.F.: résumé de 20 années de recherche. Alauda 62: 44-47.

Kimura M. 1980. A simple method for estimating evolutionary rates of base substitutions through comparative studies of nucleotide sequences. Journal of Molecular Evolution 16:111-120.

Lescroel A, Bost CA. 2006. Recent decrease in Gentoo Penguin populations at Iles Kerguelen. Antarctic Science 18: 171-174.

Lynch HL. 2013. The Gentoo Penguin (Pygoscelis papua). In: García Borboroglu PG, Boersma PD. (ed.), Biology and Conservation of the World’s penguins, University of Washington Press, Seattle U.S.A.

Miyata T. 1979. Two types of amino acid substitution in protein evolution. Journal of Molecular Evolution. 12(3):219-236

Ng PC, Henikoff S. 2003. SIFT: Predicting amino acid changes that affect protein function. Nucleic Acids Research 31(13):3812-3814

Sneath PHA. 1966. Relations between chemical structure and biological activity in peptides. Journal of Theoretical Biology. 12(2):157-195

Song WS, Jeon YJ, Namgung B, Hong M, Yoon SI. 2017. A conserved TLR5 binding and activation hot spot on flagellin. Scientific Reports 7: 40878

Świderská Z, Smidova A, Buchtova L, Bryjova A, Fabianova A, Munclinger P, Vinkler M. 2018. Avian Toll-like receptor allelic diversity far exceeds human polymorphism: an insight from domestic chicken breeds. Scientific Reports 8.

Tamura K, Nei M. 1993. Estimation of the number of nucleotide substitutions in the control region of mitochondrial DNA in humans and chimpanzees. Molecular Biology and Evolution 10:512-526.

Trathan PN, Daunt FHJ, Murphy EJ. 1996. South Georgia: An Ecological Atlas. British Antarctic Survey, Cambridge, UK.

Urbina D, Tang B, Higgs PG. 2006. The response of amino acid frequencies to directional mutation pressure in mitochondrial genome sequences is related to the physical properties of the amino acids and to the structure of the genetic code. Journal of Molecular Evolution 62(3):340-361.

Velová H, Gutowska-Ding MW, Burt DW, Vinkler M. 2018. Toll-Like Receptor Evolution in Birds: Gene Duplication, Pseudogenization, and Diversifying Selection. Molecular Biology and Evolution 35:2170-2184.

Vinkler, M., Bainová, H., Bryjová, A. Tomášek, O, Albrecht, T, Bryja, J. 2015. Characterisation of Toll-like receptors 4, 5 and 7 and their genetic variation in the grey partridge. Genetica 143(1):101-112.

Weimerskirch H, Zotier R, Jouventin P. 1988. The avifauna of the Kerguelen islands. Emu 89: 15-29.
Weir BS, Cockerham CC. 1984. Estimating F-Statistics for the Analysis of Population Structure. Evolution 38:1358-1370.
Yang ZH. 2007. PAML 4: Phylogenetic analysis by maximum likelihood. Mol Biol Evol 24:1586-1591.
Yang ZH. 1997. PAML: a program package for phylogenetic analysis by maximum likelihood. Computer Applications in the Biosciences 13:555-556.
Yang ZH, Wong WSW, Nielsen R. 2005. Bayes empirical Bayes inference of amino acid sites under positive selection. Mol Biol Evol 22:1107-1118.
Yoon SI, Kurnasov O, Natarajan V, Hong M, Gudkov AV, Osterman AL, Wilson IA. 2012. Structural basis of TLR5-flagellin recognition and signaling. Science 335(6070):859-864.
